# Efficacy of perioperative intravenous iron therapy for transfusion in orthopedic surgery: A systematic review and meta-analysis

**DOI:** 10.1371/journal.pone.0215427

**Published:** 2019-05-06

**Authors:** Hye Won Shin, Jeong Jun Park, Hyun Jung Kim, Hae Sun You, Sung Uk Choi, Mee Ju Lee

**Affiliations:** Department of Anesthesiology and Pain Medicine, College of Medicine, Korea University Anam Hospital, Seoul, Republic of Korea; Providence VA Medical Center, UNITED STATES

## Abstract

Perioperative anemia frequently occurs in patients undergoing orthopedic surgery. We aimed to evaluate the efficacy of perioperative intravenous iron therapy (IVIT) on transfusion and recovery profiles during orthopedic surgery. We searched PubMed, Embase, Cochrane, and Google Scholar for eligible clinical trials (randomized controlled trials, RCTs; case-control studies, CCSs) in comparing IVIT and no iron therapy, up to September 2018. Primary outcomes were the effects of IVIT on the proportion of patients transfused and units of red blood cells (RBCs) transfused perioperatively. Secondary outcomes were the effects of IVIT on recovery profiles, such as length of hospital stay (LOS), post-operative infection, and mortality. Subgroup analysis was performed based on iron dose (low: ≤ 300 mg, high: > 400 mg), IVIT period (pre-operative, post-operative, perioperative), and study design. We identified 12 clinical trials (4 RCTs with 616 patients and 8 CCSs with 1,253 patients). IVIT significantly reduced the proportion of patients transfused by 31% (RR, 0.69; *P* = 0.0002), and units of RBCs transfused by 0.34 units/person (MD, −0.34; *P* = 0.0007). For subgroup analysis by iron dose, low- or high-dose IVIT significantly reduced the proportion of patients transfused (RR, 0.73, *P* = 0.005; RR, 0.68, *P* = 0.008), and RBC units transfused (MD, −0.47, *P* < 0.0001; MD, −0.28, *P* = 0.04). For subgroup analysis by period, IVIT administered post-operatively significantly reduced the proportion of patients transfused (post-operative: RR, 0.60, *P* = 0.002; pre-operative: RR, 0.74, *P* = 0.06) and RBC units transfused (post-operative: MD, −0.44, *P* <0.00001; pre-operative: MD, −0.29, *P* = 0.06). For subgroup analysis by study design, IVIT decreased the proportion of patients transfused and RBC units transfused in the group of CCSs, but IVIT in the group of RCTs did not. IVIT significantly shortened LOS by 1.6 days (*P* = 0.0006) and reduced post-operative infections by 33% (*P* = 0.01). IVIT did not change mortality. Perioperative IVIT during orthopedic surgery, especially post-operatively, appears to reduce the proportion of patients transfused and units of RBCs transfused, with shorter LOS and decreased infection rate, but no change in mortality rate. These were only found in CCSs and not in RCTs due to the relatively small number of RCTs with low to high risk of bias.

## Introduction

Perioperative anemia and intraoperative blood loss in patients undergoing orthopedic surgery are risk factors for requiring red blood cell (RBC) transfusions [1]. Major orthopedic surgery, especially hip and knee arthroplasty, results in significant intraoperative bleeding [2]. The most common cause of iron deficiency anemia (IDA) is absolute iron deficiency or functional iron deficiency [3]. Functional IDA may be due to erythropoiesis blunted by inflammation in patients undergoing surgery [4]. A surge in inflammatory cytokines increases the levels of the hormone hepcidin, which impairs the absorption and recycling of iron by iron sequestration in macrophages [3]. As a regulator of iron erythropoiesis, hepcidin impairs the recycling of iron from erythrocytes, absorption of dietary iron by duodenal enterocytes, and storage of iron in hepatocytes [5]. Allogenic blood transfusion is the most frequently used treatment for perioperative anemia and bleeding; however, it is associated with the risk of disease transmission, immunomodulation, allergic reaction, infection, and cancer recurrence [6].

Perioperative iron therapy has been used to manage anemia and to reduce the rate of transfusion and transfusion-related complications [7,8]. Oral iron therapy is inexpensive and easy to administer, but the routine use of oral iron therapy is limited by its gastrointestinal side effects [9]. Previous studies using oral iron therapy have not shown any clinically relevant benefits when used to treat anemia associated with hip or knee surgeries [9,10]. Contrarily, there were several studies reporting good efficacy of intravenous iron therapy (IVIT) in increasing hemoglobin levels and reducing blood transfusion in patients with anemia [11–13]. However, for results regarding the efficacy of IVIT in transfusion, recovery profiles according to the status of disease or condition of the patient are controversial. The present systematic review and meta-analysis was restricted to clinical trials among patients undergoing orthopedic surgery.

Thus, the purpose of this meta-analysis and systematic review was to evaluate the efficacy of IVIT with respect to details of transfusion and recovery profiles, such as length of hospital stay (LOS), rate of post-operative infection, and mortality among patients undergoing orthopedic surgery.

## Materials and methods

In this meta-analysis of clinical trials, we evaluated the efficacy of perioperative IVIT during orthopedic surgery. This analysis was performed according to the recommendations of the PRISMA statement. This systematic review was registered in PROSPERO under the number CRD42018081647.

### Literature search

According to the protocol recommended by the Cochrane Collaboration, we performed a systematic literature search of clinical trials to evaluate the efficacy of perioperative IVIT for transfusion and recovery profiles during orthopedic surgery. We conducted a systematic search of databases, including PubMed, Embase, Cochrane Central, KoreaMed, and Google Scholar, to collect information on previous clinical trials involving adults (older than 19 years) up to September 2017, with no language restrictions. We then expanded the systematic literature search to September 2018. As outlined in the Supporting Information (S1 Fig), the following key words were used: “orthopedic”, “iron”, and “intravenous”.

### Study selection

Peer-reviewed clinical trials that evaluated the efficacy of perioperative IVIT for transfusion and recovery profiles during orthopedic surgery in adult patients were included in our analysis. Review articles, case reports, letters to the editor, commentaries, proceedings, laboratory studies, and other non-relevant studies were excluded. Two authors (Lee MJ and Park JJ) independently assessed the articles for compliance with the inclusion/exclusion criteria. Any disagreement was resolved though discussion or consultation with a third independent investigator (HWS).

### Data extraction and assessment of outcomes

The primary outcomes were to evaluate the effects of IVIT with respect to the proportion of patients who received transfusion and units of RBCs transfused during the perioperative period. Secondary outcomes were to evaluate the effects of IVIT with respect to recovery profiles, such as LOS, post-operative infection, and mortality.

Using standardized forms, two authors (Kim HJ and Yoo HS) independently extracted the following data: name of the first author, year of publication, type of surgery, number of patients who received transfusion, units of RBCs transfused, LOS, rate of post-operative infection, and mortality. We attempted to contact the authors of studies with insufficient or missing data. If this was impossible, we extrapolated data from the figures to obtain the target information. The values for units of RBCs transfused and LOS were converted to units per patient or days per patient, and the proportion of patients who received transfusion, the rate of post-operative infection, and the mortality were reported as the number of patients per total patients. The control group included patients who did not receive IVIT, and the intervention group included patients who received IVIT during the perioperative period.

### Assessment of bias risk

Two authors (Shin HW and Choi SU) independently evaluated the quality of clinical trials. We used the Cochrane Risk-of-Bias tool to assess the quality of randomized controlled trials (RCTs) using the following seven potential sources of bias: random sequence generation, allocation concealment, blinding of participants, blinding of outcome assessment, incomplete outcome data, selective outcome reporting, and other sources of bias [14]. In addition, we also used the Newcastle-Ottawa scale to assess the quality of non-randomized controlled studies [case-controlled studies (CCSs)] in the meta-analysis, according to three methodological aspects (selection of participants, group comparability, and outcomes), using a 9-point scale [15]. The methodology for each clinical trial was graded as high, low, or unclear to reflect either a high, low, or uncertain risk of bias, respectively.

### Statistical analysis

Statistical analysis was performed using RevMan version 5.3 (Cochrane Collaboration, London, UK). The mean difference (MD) with its 95% confidence interval (CI) was calculated for continuous variables, and the relative risk (RR) with its corresponding 95% CI was obtained for dichotomous outcome data. The overall data were collected using a Z-test. All reported *P-*values are two-sided. A two-sided *P-*value < 0.05 was considered statistically significant. Statistical heterogeneity was estimated using the *I*^2^ statistic, which was considered significant above 50%. Due to the relatively small number of clinical trials and the resulting clinical heterogeneity in our meta-analysis, the Mantel–Haenszel test or inverse-variance random-effects model was used instead of the fixed-effects model. To assess the heterogeneity of outcomes, we performed a sensitivity analysis to evaluate the influence of a single study on the overall effect estimated by excluding one study at a time. Subgroup analyses were performed based on the dose of IV iron (low dose: ≤ 300 mg and high dose: > 400 mg), the period of IVIT (preoperative, post-operative, and perioperative period), and the study design (RCTs vs. CCSs).

The presence of possible publication bias was suspected if the funnel plot was visually asymmetric or if the *P*-values were < 0.1 in an Egger's linear regression test. In such cases, a trim and fill analysis was performed to confirm publication bias.

## Results

### Literature search

**Fig 1:**
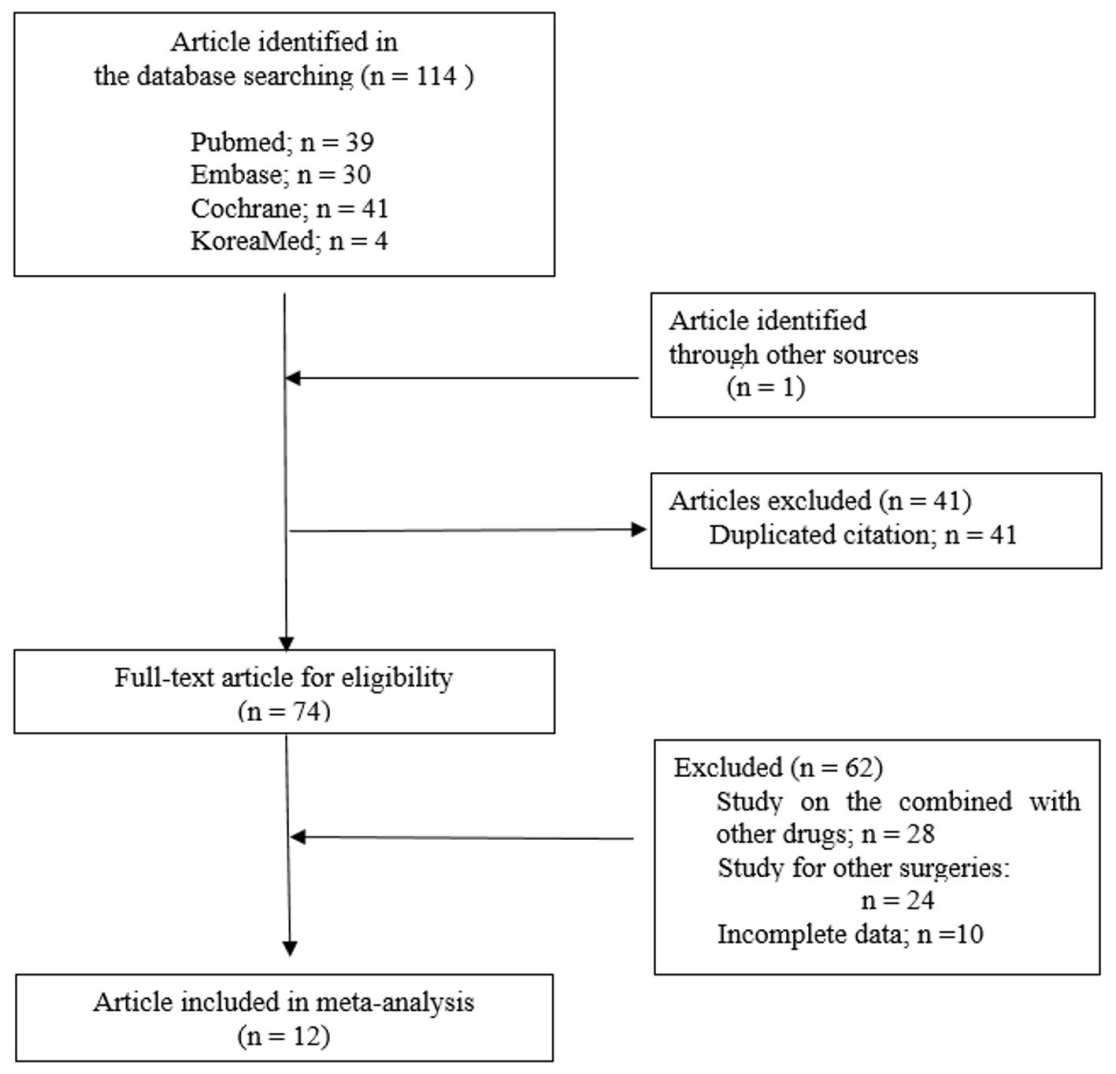
PRISMA flow diagram for the study inclusion and exclusion process.

During the initial electronic search, 115 potential clinical trials were identified (39 from PubMed, 30 from Embase, 41 from Cochrane Central, 4 from KoreaMed, and 1 from other sources), as described in Fig 1. We identified 12 studies [16–27] that involved the use of IVIT in different types of orthopedic surgeries as well as other surgeries; these studies were published between 2004 and 2018, compromising 1,869 patients. No further records were obtained from ClinicalTrials.gov or by contacting the authors.

### Study characteristics and data

We included a total of 12 clinical studies, comprising 4 RCTs with 616 patients [16–19] and 8 CCSs with 1,253 patients [20–27] (Table 1). The studies included in this review originated from four countries: Australia [17], Canada [19], Republic of Korea [20,23], and Spain [16,18,21,22,24–27] (Table 1). The patients had undergone orthopedic hip or knee surgery [16,18,20–28], and orthopedic and other surgery [17,19]. The details of IVIT were recorded according to the type of IV iron (699 patients received IV ferric carboxymaltose (FCM) [16,17,20], 806 patients received IV iron sucrose [18,19,22–27], and 364 patient received IV FCM or iron sucrose [21]), the period of IVIT (pre-operative [16,22–24,26,27], post-operative [17,19–21,25], and perioperative period [18]), and the dose of IV iron (≤ 300 mg [23,25–27] and > 400 mg [16–22,24]) (Table 1). If necessary, we extrapolated data from the figures to obtain the target information [25].

**Table 1:** Characteristics of the included studies.

**A.** Randomized controlled trials (RCTs)							
**Study**	**Country**	**Surgery**	**Groups**	**IV iron therapy period and amount**	**Number of patients**	**IV iron therapy**	**Hematologic findings of participants**
Bernabeu-Wittel	Spain	Hip fracture surgery	Control group		97	IV ferric carboxymaltose 1000 mg × 1 day	Patient with heart failure; Hb level 7.1–8.9 g/dL or < 7 g/dL
et al., 2016 [16]			Intervention group	Pre-operative	101		
				IV iron 1000 mg			
				Preoperative	97		
				erythropoietin			
Khalafallah et al., 2016 [17]	Australia	Major orthopedic surgery and other surgery	Control group		98	IV ferric carboxymaltose 1000 mg × 1 day	Hb level: 7–12 g/dL at post-operative day 1.
			Intervention group	Post-operative	103		
				IV iron 1000 mg			
Serrano-Trenas et al., 2011 [18]	Spain	Hip fracture surgery, age > 65 years	Control group		97	IV iron sucrose 200 mg/48 h × 3 doses	No comment.
			Intervention group	Perioperative	99		
				IV iron 600 mg			
Karkouti et al., 2006 [19]	Canada	Orthopedic surgery or cardiac surgery	Control group		10	IV iron sucrose 200 mg/day × 3 doses	Hb level 7–9 g/dL at post-operative day 1.
			Intervention group	Post-operative	11		
				IV iron 600 mg			
				Post-operative	10		
				IV iron + erythropoietin			
**B.** Case-controlled studies (CCSs)							
**Study**	**Country**	**Surgery**	**Groups**	**IV iron therapy period and amount**	**Number of patients**	**IV iron therapy**	**Hematologic findings of participants**
Kim et al., 2018 [20]	Republic of Korea	Orthopedic hip surgery	Control group		150	IV ferric carboxymaltose 1000 mg × 1 day	Hb level: < 10 g/dL or Hb fall
							> 3 g/dL
			Intervention group	Post-operative	150		at post-operative day 1.
				IV iron 1000 mg			
Munoz et al., 2014 [21]	Spain	Total lower limb arthroplasty	Control group		182	IV iron sucrose 200 mg × 3 doses	Hb level: <10 g/dL at post-operative day 1.
						(n = 134)	
						or	
			Intervention group	Post-operative	182	IV iron ferric carboxymaltose 600 mg x 1 dose (n = 48)	
				IV iron 600 mg			
Blanco Rubio et al.,	Spain	Hip fracture surgery	Control group		63	IV iron sucrose 200 mg × 3 doses	No comment.
2013 [22]			Intervention group	Pre-operative	57		
				IV iron 600 mg			
Bae et al., 2010 [23]	Republic of Korea	Knee arthroplasty	Control group		30	IV iron sucrose 200 mg × 1 day and 100 mg × 1 day	No comment.
			Intervention group	Pre-operative	30		
				IV iron 300 mg			
Gonzalez-Porras et al., 2009 [24]	Spain	Total hip replacement or total knee replacement surgery	Control group		80	IV iron sucrose 200 mg/week × 4 doses	Hb level >10 g/dL in preoperative laboratory findings
			Intervention group	Pre-operative	49		
				IV iron 800 mg			
				Pre-operative	145		
				oral iron			
				Pre-operative blood donation	20		
				+ oral iron			
				Pre-operative erythropoietin	9		
				+ IV iron			
Munoz et al., 2006 [25]	Spain	Total hip replacement surgery	Control group		22	IV iron sucrose 100 mg/day × 3 doses	No comment.
			Intervention group	Post-operative	24		
				IV iron 300 mg			
Cuenca et al., 2005 [26]	Spain	Displaced subcapital hip fracture repair surgery, age > 65 years	Control group		57	IV iron sucrose 100 mg/day × 2 doses;	No comment.
			Intervention group	Pre-operative	20	if Hb level <12 g/dL, 1 dose	
				IV iron 200 mg			
Cuenca et al., 2004 [27]	Spain	Pertrochanteric hip fracture repair surgery, age > 65 years	Control group		102	IV iron sucrose 100 mg/day × 2 doses;	No comment.
			Intervention group	Pre-operative	55	if Hb level <12 g/dL, 3 doses	
				IV iron 200 mg			

Hb, hemoglobin; IV, intravenous

### Bias risk assessment

The results of an assessment of the bias risk and methodology of the included studies are summarized in Table 2. All RCTs used randomization with concealed allocation [16–19]. Two RCTs showed a high risk of bias, particularly due to the absence of or unreported blinding of participants or outcome assessments [17,18].

**Table 2:** Bias risk assessment and methodology of the meta-analysis.

**A.** Cochrane risk of bias assessment tool for assessing the quality of randomized controlled studies										
**References**	**Random sequence generation**	**Allocation concealment**	**Blinding of participants**	**Blinding of outcome assessment**	**Incomplete outcomes**	**Selective data reporting**	**Other biases**			
Bernabeu-Wittel et al., 2016 [16]	Low risk	Low risk	Low risk	Low risk	Low risk	Low risk	Unclear risk			
Khalafallah et al., 2016 [17]	Low risk	Low risk	High risk	High risk	Low risk	Low risk	Low risk			
Serrano-Trenas et al., 2011 [18]	Low risk	Low risk	High risk	High risk	Low risk	Low risk	Low risk			
Karkouti et al., 2006 [19]	Low risk	Low risk	Low risk	Low risk	High risk	Low risk	Unclear risk			
**B.** Outcome of assessment of the quality of case-control studies using the Newcastle-Ottawa scale										
	**Selection**				**Compatibility**		**Outcomes**			**Total score**
	Representativeness of the exposed cohort	Selection of non-exposed cohort	Ascertainment of exposure	Outcome not presented at the start	Compatibility;	Compatibility;	Assessment of outcome	Lost to follow-up	Adequate follow-up	
					age and sex	additional factors				
Kim et al., 2018 [20]	*	*	*	*	*	*	–	*	*	8/9
Munoz et al., 2014 [21]	*	*	*	*	*	*	–	*	*	8/9
Blanco Rubio et al., 2013 [22]	*	*	*	*	*	*	–	*	*	8/9
Bae et al., 2010 [23]	*	*	*	*	*	*	–	*	*	8/9
Gonzalez-Porras et al., 2009 [24]	*	*,	*	*	*	*	–	*	*	8/9
Munoz et al., 2006 [25]	*	–	*	*	*	*	–	*	*	7/9
Cuenca et al., 2005 [26]	*	–	*	*	*	*	–	*	*	7/9
Cuenca et al., 2004 [27]	*	–	*	*	*	*	–	*	*	7/9

A single asterisk (*) indicates 1 score, and dash (–) indicates 0 score.

All CCSs showed low risk for bias, with scores of more than 7 out of 9 points on the Newcastle-Ottawa scale [20–27]. Four CCSs included retrospective data in the control group [20,25–27]. All CCSs assessed the outcomes using unblinded assessment or unknown measurement methods.

### Publication bias

We observed a funnel plot for every comparison. There was funnel asymmetry for publication bias in all items (S2 Fig). Egger’s linear regression method was used to evaluate the publication bias for the following comparisons (>10 included studies for a comparison): the proportion of patients who received transfusion (coef. −1.24; 95% CI, −3.18 to 0.69; *P* = 0.180), the units of RBCs transfused (coef. 0.80; 95% CI, −0.97 to 2.58; *P* = 0.333), LOS (coef. −1.85; 95% CI, −3.37 to −0.33; *P* = 0.022), and rate of post-operative infection (coef. −2.05; 95% CI, −4.27 to 0.17; *P* = 0.066). The results are shown in the Supporting Information (S2 Fig). Therefore, publication bias was not noted for the proportion of patients who received transfusion and units of RBCs transfused.

To compare *P*-values < 0.1 derived by Egger’s method, we performed a trim and fill analysis. We observed no changes in statistical significance for LOS (MD, −1.48; 95% CI, −2.34 to −0.62; *P* = 0.001) and rate of post-operative infection (RR, 0.63; 95% CI, 0.41 to 0.96; *P* = 0.032). Therefore, publication bias was noted for LOS and rate of post-operative infection (S2 Fig).

### Results of meta-analysis

#### Patients who received transfusion (%)

**Fig 2:**
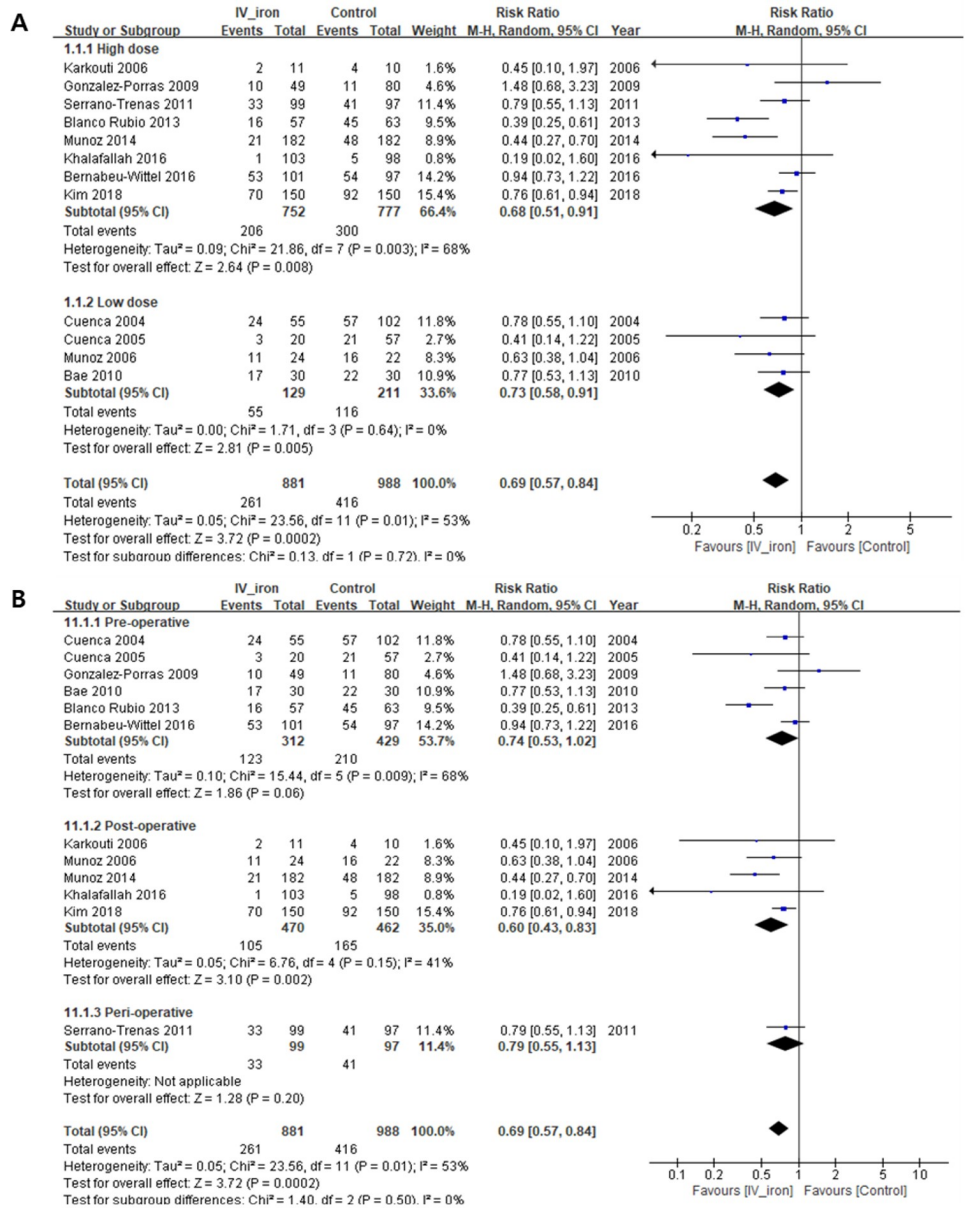
Forest plot demonstrating the proportion of patients who received transfusion (%). Subgroup analysis according to (A) the iron dose and (B) period of intravenous iron therapy (IVIT) administration. CI, confidence interval; *I*^2^, statistical heterogeneity.

IVIT significantly decreased the proportion (%) of patients who received transfusion (RR, 0.69; 95% CI, 0.57 to 0.84, *I*^2^ = 53%; *P* = 0.0002). In the sub-group analysis by iron dose, IVIT decreased the proportion of patients who received transfusion with high-dose (RR, 0.68, *I*^2^ = 68%; *P* = 0.008) and low-dose (RR, 0.73, *I*^2^ = 0%; *P* = 0.005) IVIT, with no subgroup differences (*I*^2^ = 0%; *P* = 0.72) (Fig 2A and Table 3). In the sub-group analysis by period, IVIT in the post-operative period decreased the proportions of patients who received transfusion (RR, 0.60, *I*^2^ = 41%; *P* = 0.002), but IVIT in the pre-operative (RR, 0.74, *I*^2^ = 68%; *P* = 0.06) and perioperative (RR, 0.79, *I*^2^ = not applicable; *P* = 0.20) periods did not, with no subgroup differences (*I*^2^ = 0%; *P* = 0.50) (Fig 2B). In the subgroup analysis by study design, IVIT decreased the proportion of patients who received transfusion in the group of CCSs (RR, 0.65, *I*^2^ = 58%; *P* < 0.00005), but IVIT in the group of RCTs (RR, 0.84, *I*^2^ = 15%; *P* = 0.20) did not (S3 Fig). Sensitivity analysis did not change the overall significance for the proportion of patients who received transfusion.

**Table 3:** Results of meta-analysis for the proportion of patients who received transfusion and units of RBCs transfused. Sub-group analysis according to the dose of intravenous (IV) iron (low dose: ≤ 300 mg and high dose: > 400 mg), the period of intravenous iron therapy (IVIT) (pre-operative, post-operative, and perioperative periods), and the study design (RCTs vs. CCSs).

Outcome	Subgroup	design of studies	Reference	RR or MD	95% CI		*I*^2^	*P*-value	Sub-group differences	
									*I*^2^	*P*-value
**[Subgroup analysis] Low dose vs. High dose**										
Patients who received transfusion (%)	Low dose	RCTs	No study	−	−		−	−	0%	0.72
		CCSs	[23,25–27]	0.73	0.58, 0.91		0%	0.005*		
		All studies		0.73	0.58, 0.91		0%	0.005*		
	High dose	RCTs	[16–19]	0.84	0.65, 1.09		15%	0.20		
		CCSs	[20–22,24]	0.63	0.39, 1.00		79%	0.05		
		All studies		0.68	0.51, 0.91		68%	0.008*		
RBCs transfusion (U/person)	Low dose	RCTs	No study	−	−		−	−	0%	0.80
		CCSs	[23,25–27]	−0.47	−0.71, −0.24		0%	< 0.0001 *		
		All studies		−0.47	−0.71, −0.24		0%	< 0.0001 *		
	High dose	RCTs	[16,18,19]	−0.11	−0.33, 0.10		0%	0.31		
		CCSs	[20–22,24]	−0.40	−0.78, −0.02		93%	0.04*		
		All studies		−0.28	−0.54, −0.02		89%	0.04*		
**[Subgroup analysis] Pre-operative vs. Post-operative vs. Perioperative**										
Patients who received transfusion (%)	Pre-operative	RCTs	[16]	0.94	0.73, 1.22	NA		0.65	0%	0.50
		CCSs	[22–24,26,27]	0.68	0.46, 1.02	66%		0.06		
		All studies		0.74	0.53, 1.02	68%		0.06		
	Post-operative	RCT	[17,19]	0.34	0.10, 1.15	0%		0.08		
		CCSs	[20,21,25]	0.62	0.44, 0.88	59%		0.007*		
		All studies		0.60	0.43, 0.83	41%		0.002*		
	Perioperative	RCTs	[18]	0.79	0.55, 1.13	NA		0.20		
		CCSs	No study	−	−	−		−		
		All studies		0.79	0.55, 1.13	NA		0.20		
RBCs transfusion (U/person)	Pre-operative	RCTs	[16]	−0.02	−0.40, 0.36	NA		0.92	67%	0.03
		CCSs	[22–24,26,27]	−0.36	−0.73, 0.02	81%		0.06		
		All studies		−0.29	−0.60, 0.01	76%		0.06		
	Post-operative	RCT	[19]	−0.23	−0.66, 0.20	NA		0.29		
		CCSs	[20,21,25]	−0.44	−0.46,−0.42	0%		< 0.00001*		
		All studies		−0.44	−0.46,−0.42	0%		< 0.00001*		
	Perioperative	RCTs	[18]	−0.11	−0.44, 0.22	NA		0.51		
		CCSs	No study	−	−	−		−		
		All studies		−0.11	−0.44, 0.22	NA		0.51		

RCTs, randomized controlled trials; CCSs, case-controlled studies; RR, relative risk (%); MD, mean difference (min); CI, confidence interval; *I*^2^: statistical heterogeneity; RBCs, red blood cells; NA, not applicable due to single study; −, no study.

* *P* < 0.05.

#### Units of RBCs transfused during the perioperative period (U/person)

**Fig 3:**
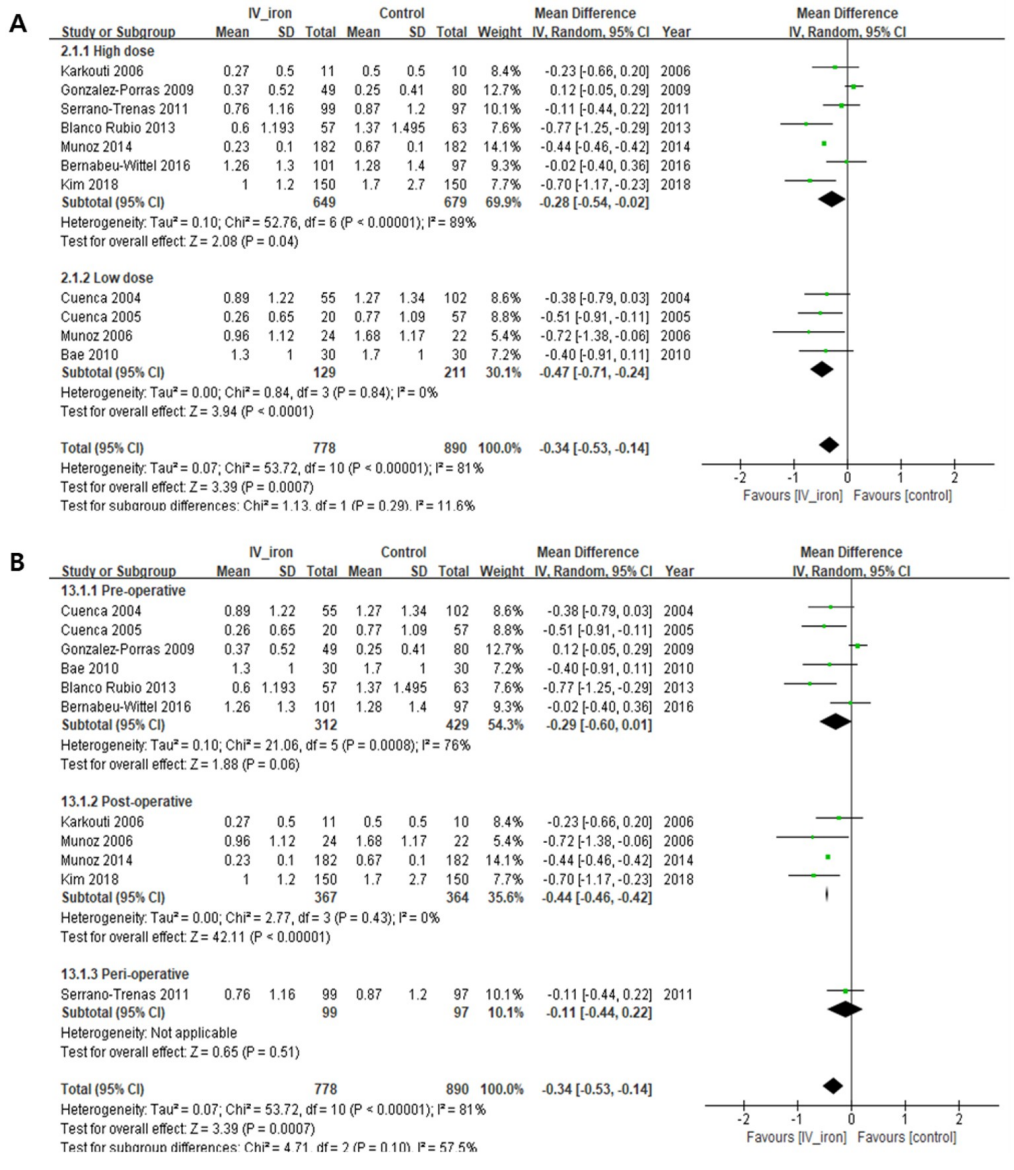
Forest plot showing the units of RBCs transfused (U/patient). Subgroup analysis according to (A) iron dose and (B) period of intravenous iron therapy (IVIT). SD, standard deviation; CI, confidence interval; *I*^2^, statistical heterogeneity.

IVIT significantly reduced the units of RBCs transfused (U/person) (MD, −0.34; 95% CI, −0.53 to −0.14, *I*^2^ = 81%; *P* = 0.0007). In the sub-group analysis by iron dose, IVIT reduced the units of RBCs with high-dose (MD, −0.28, *I*^2^ = 89%; *P* = 0.04) and low-dose (MD, −0.47, *I*^2^ = 0%; *P* < 0.0001) IVIT, with no subgroup differences (*I*^2^ = 11.6%, *P* = 0.29) (Fig 3A and Table 3). In the sub-group analysis by IVIT period, IVIT given post-operatively reduced the units of RBCs (MD, −0.44, *I*^2^ = 0%; *P* < 0.00001), but IVIT given pre-operatively (MD, −0.29, *I*^2^ = 76%; *P* = 0.06) and perioperatively (MD, −0.11, *I*^2^ = not applicable; *P* = 0.51) did not reduce the units of RBCs, with no significant subgroup differences (*I*^2^ = 57.5%, *P* = 0.10) (Fig 3B and Table 3). In the subgroup analysis by study design, IVIT reduced the units of RBCs transfused in the group of CCSs (RR, −0.43, *I*^2^ = 84%; *P* = 0.0005), but IVIT in the group of RCTs (RR, −0.11, *I*^2^ = 0%; *P* = 0.31) did not (S3 Fig). Based on the sensitivity analysis, overall significance in the proportion of patients who received transfusion did not change.

#### Recovery profiles

**Fig 4:**
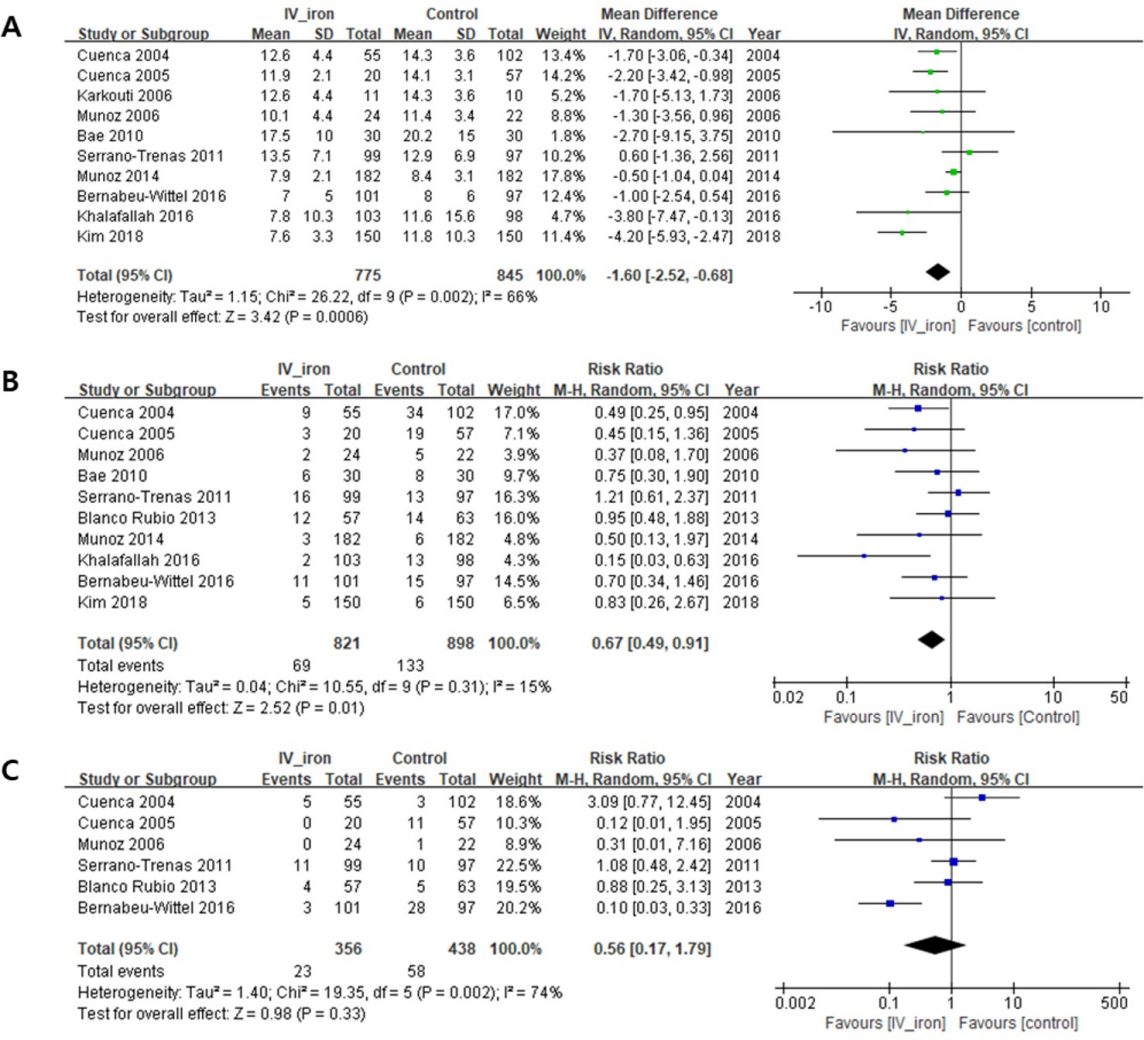
**Recovery profiles: (A) length of hospital stay (days), (B) post-operative infections (%), (C) mortality (%).** SD, standard deviation; CI, confidence interval; *I*^2^, statistical heterogeneity.

IVIT therapy shortened LOS (days) (MD, −1.60; 95% CI, −2.52 to −0.68; *I*^2^ = 66%; *P* = 0.0006) and reduced the rate of post-operative infection (%) (RR, 0.67; 95% CI, 0.49 to −0.91; *I*^2^ = 15%; *P* = 0.01), in comparison with the control group. However, IVIT did not change the mortality rate (%) (RR, 0.56; 95% CI, 0.17 to 1.79; *I*^2^ = 74%; *P* = 0.33) (Fig 4). In the subgroup analysis by study design, IVIT in the group of CCSs shortened LOS (MD, −1.91; *I*^2^ = 76%; *P* = 0.002) and reduced the rate of post-operative infection (%) (RR, 0.64; *I*^2^ = 0%; *P* = 0.01), but IVIT in the group of RCTs (MD, −0.98; *I*^2^ = 38%; *P* = 0.21 / RR, 0.61, *I*^2^ = 71%; *P* = 0.31) did not (S3 Fig).

#### Hemoglobin concentration and ferritin

We also conducted a meta-analysis to analyze the concentrations of hemoglobin and ferritin between IVIT and control groups (S4 File). For hemoglobin concentrations, there were no significant changes at admission, postoperative day 1, day 7, and around day 60. For ferritin concentration, there were no significant changes at baseline (before IVIT), postoperative day 1 and day 7; however, there were significant changes at post-operative week 4 and day 60. It should be noted that a limitation of the analysis for ferritin concentration was that we included only two studies with large standard deviations.

## Discussion

The present systematic review and meta-analysis of 12 clinical trials indicated that perioperative IVIT decreased the proportion of patients transfused by 31% and the RBCs transfused by 0.34 units among patients undergoing orthopedic surgery. IVIT also shortened LOS by 1.60 days and reduced the rate of post-operative infections by 33%, with no change in the mortality rate. IVIT administered in the post-operative period significantly reduced the proportion of patients who received transfusion and units of RBCs, as compared with the control group. However, the above results were only present in CCSs and not in RCTs.

Functional IDA commonly occurs during major surgery, despite normal iron stores in the bone marrow, because iron is unavailable for erythropoiesis owing to the alteration of its release from macrophages and its incorporation into transferrin [3,5,29,30]. Andrew et al. [31] reported that pre-operative iron therapy could prevent post-operative anemia in patients with functional IDA. Generally, perioperative anemia during orthopedic surgery is most commonly treated with allogenic blood transfusion. Currently, the alternative to transfusion is the administration of iron, which may contribute to rapid and safe recovery from erythroid mass.

Oral iron supplementation is the first-line treatment for most patients because of its convenience and low cost. However, oral iron therapy for functional IDA might not be useful due to ineffective gastrointestinal absorption and side effects [19,26]. Compared with oral iron, IVIT is more efficacious in IDA, for the rapid delivery of iron without severe adverse effects during trauma or surgery [32,33]. IV iron directly binds transferrin in plasma [23], and the erythropoietic effect of IV iron is increased about 5 times and lasts for 7–10 days [25]. Some meta-analyses have examined the efficacy of perioperative iron therapy. Yang et al. conducted a meta-analysis [34] in elderly patients undergoing hip or knee surgery (5 studies with oral iron therapy, 1 study with IVIT) in comparison with a control group who did not receive iron therapy. Hemoglobin levels showed a greater increase in the iron therapy group than in the controls. However, there were no significant changes in the details for transfusion, LOS, morbidity, and infection rate between the iron therapy and control groups. Hallet et al. performed a meta-analysis [35] in patients undergoing gastrointestinal surgery (2 studies with oral iron therapy, 2 studies with IVIT); they found that the rate of transfusion was lower in the iron therapy group than in the control group, but there was no significant difference in the units of RBCs transfused. Previously reported systematic reviews of iron therapy have performed analyses with respect to oral or IV iron therapy in patients with varying states of disease or surgeries [36]. We restricted the included studies for this systematic review, to confirm the efficacy of IVIT for patients with similar conditions of orthopedic surgeries who had relatively large blood loss, according to details of transfusion and recovery profiles.

Different IV iron formulations are available. IV iron sucrose or FCM was used in studies included in this meta-analysis. IV iron formulation is composed of an iron core and carbohydrate shell to prevent uncontrolled iron release into the circulation. Patients who received IVIT may be at risk for anaphylaxis when iron dextran is used. Adverse effects have been reported, such as gastrointestinal side effects for oral iron, and hypotension, anaphylaxis, infection, hypophosphatemia, oxidative stress, and mortality for IV iron [32]. In a large systematic review of IVIT safety by Avni et al., including 103 RCTs and 10,390 patients [37], IVIT was not associated with an increased risk of severe cardiovascular, respiratory, and neurologic adverse effects. There was neither an increase in adverse effects that required discontinuation nor an increase in mortality. However, infusion reactions at the IV site occur with IV iron [37]. Iron sucrose can be safely administered as a bolus over 2 minutes or as a short infusion for doses up to 300 mg [33]. FCM is a non-dextran IV iron formulation with a carboxymaltose shell, which minimizes the release of free iron with greater iron delivery to tissues [12,38]. FCM can be administered as a single infusion over 15 minutes and at large iron doses (up to 750 mg in the United States and up to 1000 mg in the European Union) [39]. The iron dose of FCM is sufficient for the restoration of deficits in iron stores during the perioperative period. In the future, FCM may be considered the first-line therapy in patients undergoing orthopedic surgery [12,33,40]. Recent guidelines for transfusion during surgery [13,41] recommend perioperative IVIT in patients with functional IDA who are expected to have large blood loss during surgery.

Based on this meta-analysis of CCSs, we found that the incidence of post-operative infection is decreased, and LOS is shortened after IVIT. A previous study showed an increased risk for infection after IVIT because free iron is a pro-oxidant and a micronutrient for bacterial growth in vitro [42]. A meta-analysis by Litton et al. [11] reported that IVIT was associated with a significant increase in the risk of infection. On the contrary, Maretty et al. [43] reported that FCM significantly increased animal weight, and enhanced reticulocytosis as well as recovery in malaria-infected animals. A systematic review by Avni et al. [33] reported that there was no increased risk of infection. For recovery profiles, a decreased number of transfusions and post-operative infections might influence LOS.

Generally, the reported adverse effects of IVIT include hot flushes, chest tightness, headache, nausea, vomiting, mild fever, arthralgia, and anaphylaxis associated with free iron toxicity [44]. As for the studies included in this meta-analysis [16, 18, 21, 22, 24–26], there were no severe adverse effects attributable to IVIT. With the use of newer IV iron preparations, the decrease in adverse effects may be related to lower concentrations of free iron [37]. In addition, there were no changes in hemoglobin concentration between groups in our review, due to the replacement of transfusion for hemodynamic stability during the perioperative period. In evaluating IDA, ferritin represents the iron stores in the body [1]. In our review, ferritin levels were increased at postoperative week 4 and day 60, but the interpretation of ferritin results is limited by the small number of included studies and the large standard deviation of the data. Ferritin also acts as an acute-phase reactant, and its levels may be increased by inflammation during the perioperative period [17]. Additional studies using biomarkers of IDA during surgery are required.

In Perelman et al.’s [36] systematic review using the results of RCTs for the efficacy of post-operative (oral or intravenous) iron therapy in clinical outcomes (hemoglobin levels, patient-centered quality of life, blood transfusion requirement) following surgery, there were no evidence to support the routine use of post-operative iron therapy in surgical patients. In the subgroup analysis by period, we found that IVIT administered in the post-operative period significantly reduced the proportion of patients transfused and units of RBCs, as determined based on comparisons with these values in the control group, which did not receive IVIT. However, there was no significant change for IVIT in the pre-operative period. These results were only found in CCSs and not in RCTs due to the relatively small number of RCTs. IV iron can be utilized and released immediately by macrophages to become readily available for erythropoiesis. Inflammation during the perioperative period increases hepcidin levels, leading to impaired intestinal iron absorption and lower serum iron and transferrin saturation, which contribute to functional IDA. Patients with inflammation can benefit from perioperative IVIT by overcoming hepcidin-mediated blocking of iron absorption and recycling and iron-restricted erythropoiesis [36].

The strengths of this meta-analysis include a systematic literature search conducted with no restriction of language or type of publication. The inclusion of RCTs and CCSs permitted a thorough and inclusive review of interventions for orthopedic surgery. We also conducted a review for secondary outcomes, such as post-operative infection and morbidity.

However, our review has several sources of potential bias and a moderate-to-high level of heterogeneity in the outcomes. First, the publication bias exists in the parameters of LOS and post-operative infection. Second, for 12 clinical trials comprising 4 RCTs and 8 CCSs, 2 RCTs were considered to have a high level of bias in the blinding of participants or outcome assessments, and 4 CCSs used retrospective data in the control group. All CCSs assessed the outcomes using unblinded or unknown methods. Third, 3 CCSs did not clearly identify the selection of a control group. Fourth, 4 included clinical trials had overlapping research groups, which may add further bias to the meta-analysis. Finally, the methods and results of the included trials comprised varying types and doses of IV iron preparations, and transfusion periods.

## Conclusions

In the systematic review using 8 CCSs, perioperative IVIT, especially post-operatively, is an effective alternative to transfusion and revealed good recovery profiles during orthopedic surgery. However, based on our meta-analysis using the results of 4 RCTs, we could not identify the definitive effect of IVIT on the profiles regarding transfusion and recovery. The small numbers of RCTs for all parameters are inadequate for satisfactory statistical analysis. Therefore, we recommend that there be large, prospective, well-designed RCTs to confirm the efficacy of perioperative IVIT in patients with functional IDA during major surgery.

## Supporting information

S1 Fig Search strategy. (PDF)

S2 Fig Egger’s linear regression and Trim-and-fill analysis to assess publication bias. (PDF)

S3 Fig Comparison between the groups of randomized controlled trials (RCTs) vs. case-controlled studies (CCSs). (PDF)

S4 Fig Comparison of hemoglobin and ferritin levels between the intravenous iron therapy (IVIT) and control groups. (PDF)

S1 Table PRISMA checklist. (PDF)
