# Supplementary material for: Efficacy of perioperative intravenous iron therapy for transfusion in orthopedic surgery: A systematic review and meta-analysis
Source: PLoS One. 2019 May 6;14(5):e0215427. doi: 10.1371/journal.pone.0215427 (PMC6502310; doi:10.1371/journal.pone.0215427)
Supplement: S1 Fig — (PDF) [file pone.0215427.s001.pdf]

Supporting Information

S 1. The search strategy

Pubmed (10-Sep-18)

1. Search "Orthopedics"[Mesh] 19,539
2. Search (Orthopedic[tiab] OR Orthopedics[tiab] OR bone[tiab] OR orthopaedic[tiab]) AND (surgery[tiab] OR surgeries[tiab] OR operation[tiab] OR operations[tiab] OR procedure[tiab] OR procedures[tiab] OR surgical[tiab]) 145,130
3. Search "Orthopedic Procedures"[Mesh:NoExp] 23,099
4. Search ("Acetabuloplasty"[Mesh] OR "Arthrodesis"[Mesh] OR "Arthroplasty"[Mesh] OR "Bone Lengthening"[Mesh] OR "Bone Transplantation"[Mesh] OR "Cementoplasty"[Mesh] OR "Fracture Fixation"[Mesh] OR "Osteotomy"[Mesh]) 194,249
5. Search ((Orthopedic[tiab] OR Orthopaedic[tiab]) AND (Surgical[tiab] OR surgery[tiab]) AND (Procedures[tiab] OR Procedure[tiab])) OR Acetabuloplasty[tiab] OR Arthrodesis[tiab] OR Arthroplasty[tiab] OR Bone Lengthening[tiab] OR Bone Transplantation[tiab] OR Cementoplasty[tiab] OR Fracture Fixation[tiab] OR Osteotomy[tiab] OR Hip surgery[tiab] OR hip surgeries[tiab] OR hip fracture[tiab] OR hip fractures[tiab] OR Femur surgery[tiab] OR femur sugeries[tiab] 113,765
6. Search (#1 OR #2 OR #3 OR #4 OR #5) 363,525
7. Search blood transfusion[mesh] 80,865
8. Search blood transfusion[tiab] OR blood transfusions[tiab] OR transfusion[tiab] OR transfusions[tiab] 103,497
9. Search (#7 OR #8) 139,925
10. Search intravenous injections[mesh] OR intravenous[tiab] OR parenteral[tiab] 352931
11. Search "Iron"[Mesh] OR "Iron Compounds"[Mesh] OR "Hemosiderin"[Mesh] OR "Transferrin"[Mesh] OR ferric compounds[mesh] OR ferrous compounds[mesh] OR iron carbonyl compounds[mesh] 156,548
12. Search iron[tiab] OR Ferritin[tiab] OR Ferritins[tiab] OR Isoferritin[tiab] OR ferric compounds[tiab] OR ferric compound[tiab] OR ferrous compunds[tiab] OR hemosiderin [tiab] OR transferrin [tiab] 196589
13. Search (#11 OR #12) 263,714
14. Search (#6 AND #9 AND #10 AND #13) 79
15. Search ((Therapy/Broad[filter]) AND (((orthopedics OR orthopedic OR orthopaedic OR orthopaedics) AND (surgery OR surgical OR surgeries)) AND (transfusion OR transfusions) AND (intravenous OR parenteral) AND (iron OR ferritin OR ferritins OR isoferritin OR transferrin))) 44
16. Search (#14 OR #15) 82
17. Search (animals [mh:noexp] OR animal experimentation[mesh] OR animal models[mesh] 6,269,222
18. Search animals[mesh] AND human[mesh] 1,726,5971
19. Search (#17 NOT #18) 4,463,474
20. Search (#16 NOT #19) 82
21. Search (#14 OR #15) Filters: Humans 71
22. Search (#20 OR #21) 82

23. Search (clinical trials as topic[mesh] OR clinical trial[pt] OR Controlled Clinical Trials as Topic [Mesh] OR controlled clinical trial [pt] OR Randomized Controlled Trials as Topic[Mesh] OR randomized controlled trial [pt] OR Multicenter Study[pt] OR random allocation [mesh] OR Double Blind [tw] OR Random Allocat* OR randomized controlled trial OR randomised controlled trial OR controlled clinical trial* OR controlled trial* OR clinical trial* OR Double blind* OR single blind* OR triple blind* OR double mask* OR single mask* OR triple mask* OR randomly OR randomized OR randomised OR randomization OR randomisation OR placebo [tw] OR multicenter*[tw]) NOT (Editorial[pt] OR Letter[pt] OR Case Reports[pt] OR Comment[pt] OR Historical Article[ptyp] OR case report* OR Historical Article* OR Letter*) 1,708,968
24. Search Epidemiologic Studies[Mesh:NoExp] OR Case-Control Studies[Mesh] OR Cohort Studies[Mesh] OR Cross-Sectional Studies[Mesh] OR Seroepidemiologic Studies[Mesh] OR comparative study[pt] OR adverse effect* OR Case control* OR cohort[tiab] OR cohort stud* OR cohort analy* OR compared group* OR concurrent stud* OR Cross sectional OR epidemiologic stud* OR epidemiological stud* OR Follow up stud* OR Follow-up stud* OR Incidence Stud* OR Longitudinal[tw] OR multivariate[tiab] OR observational stud* OR prospective stud* OR Retrospective stud* OR risk factors[mesh] 5,799,070
25. Search (#23 OR #24) 6,656,352
26. Search (#22 AND #25) 59
27. Search (#22 AND #25) Filters: Adult: 19+ years 36
28. Search (elderly OR older OR old OR adult OR adults OR elder OR young OR grown-up OR "grown up" OR mature OR grown OR aged OR "middle aged") 8,344,927
29. Search (#26 AND #28) 39
30. Search (#27 OR #29) 39

EMBASE (10-Sep-18)

1. ‘orthopedic surgery’/exp 445,451
2. (orthopedic:ti,ab OR orthopedics:ti,ab OR bone:ti,ab OR orthopaedic:ti,ab) AND (surgery:ti,ab OR surgeries:ti,ab OR operation:ti,ab OR operations:ti,ab OR procedure:ti,ab OR procedures:ti,ab OR surgical:ti,ab) 194,711
3. (orthopedic:ti,ab OR orthopaedic:ti,ab) AND (surgical:ti,ab OR surgery:ti,ab) AND (procedures:ti,ab OR procedure:ti,ab) OR acetabuloplasty:ti,ab,kw OR arthrodesis:ti,ab,kw OR arthroplasty:ti,ab,kw OR 'bone lengthening':ti,ab,kw OR 'bone transplantation':ti,ab,kw OR cementoplasty:ti,ab,kw OR 'fracture fixation':ti,ab,kw OR osteotomy:ti,ab,kw OR 'hip surgery':ti,ab,kw OR 'hip surgeries':ti,ab,kw OR 'hip fracture':ti,ab,kw OR 'hip fractures':ti,ab,kw OR 'femur surgery':ti,ab,kw OR 'femur sugeries':ti,ab,kw 137463
4. 1 OR 2 OR 3 593,277
5. ‘blood transfusion’/exp 172,957
6. 'blood transfusion':ti,ab OR 'blood transfusions':ti,ab OR transfusion:ti,ab,kw OR transfusions:ti,ab,kw 158,818
7. 5 OR 6 233,157

8. intravenous:ti,ab OR parenteral:ti,ab 407,862
9. 'iron'/exp OR 'carbonyl iron'/exp OR 'ferritin'/exp OR 'isoferritin'/exp OR 'ferric ion'/exp OR 'ferrous ion'/exp OR 'hemosiderin'/exp OR 'transferrin'/exp 214,346
10. iron:ti,ab OR ferritin:ti,ab OR ferritins:ti,ab OR isoferritin:ti,ab OR ‘ferric compounds:ti,ab OR ‘ferrous compounds’:ti,ab OR hemosiderin:ti,ab OR transferrin:ti,ab 239,017
11. 9 OR 10 315,587
12. 4 AND 7 AND 8 AND 11 148
13. 4 AND 7 AND 5 AND [humans]/lim 131
14. 4 AND 7 AND 8 AND [humans]/lim or [randomized controlled trial]/lim 35
15. 'clinical trial'/de OR 'randomized controlled trial'/de OR 'randomization'/de OR 'single blind procedure'/de OR 'double blind procedure'/de OR 'crossover procedure'/de OR 'placebo'/de OR 'prospective study'/de OR (('randomi?ed controlled' NEXT/1 trial*):ti,ab,de) OR rct:ti,ab,de OR 'randomly allocated':ti,ab,de OR 'allocated randomly':ti,ab,de OR 'random allocation':ti,ab,de OR ((allocated NEAR/2 random):ti,ab,de) OR ((singl* NEAR/1 blind*):ti,ab,de) OR ((double NEAR/1 blind*):ti,ab,de) OR (((treble OR triple) NEAR/1 blind*):ti,ab,de) OR ((cross NEXT/1 over*):ti,ab,de) OR placebo*:ti,ab,de OR crossover*:ti OR allocat*:ti OR random*:ti,ab,kw 2,643.,516
16. 'case control study'/de OR 'cohort analysis'/de OR 'comparative study'/de OR 'cross-sectional study'/de OR 'longitudinal study'/de OR 'observational study'/de OR 'prospective study'/de OR 'retrospective study'/de OR 'cohort study':ti,ab OR 'cohort studies':ti,ab OR 'cohort analyses':ti,ab OR 'cohort analysis':ti,ab OR 'cohort analytic':ti,ab OR 'follow up study':ti,ab OR 'follow up studies':ti,ab OR 'observational study':ti,ab OR 'observational studies':ti,ab OR longitudinal:ti,ab OR 'retrospective study':ti,ab OR 'retrospective studies':ti,ab OR 'cross sectional':ti,ab OR 'epidemiologic studies':ti,ab OR 'prospective study':ti,ab OR 'prospective studies':ti,ab OR 'comparative study':ti,ab OR 'risk factors'/de OR cohort:ti,ab OR 'compared groups':ti,ab OR 'case control':ti,ab OR 'epidemiologic study':ti,ab OR 'incidence study':ti,ab OR 'incidence studies':ti,ab OR 'concurrent study':ti,ab OR 'concurrent studies':ti,ab 3,956,823
17. 15 OR 16 5,717,798
18. 13 AND 17 86
19. 14 OR 18 86
20. 19 AND ([adult]/lim OR [aged]/lim OR [middle aged]/lim OR [very elderly]/lim OR [young adult]/lim) 42
21. elderly OR older OR old OR adult OR adults OR elder OR young OR 'grown up' OR mature OR grown OR aged OR 'middle aged' 9,638,594
22. 19 AND 21 46
23. 20 OR 22 46
24. 23 AND ('Article'/it OR 'Article in Press'/it) 30

Cochrane (10-Sep-18)

#1. MeSH descriptor: [orthopedics] explode all trees 325

#2. ((Orthopedic or Orthopedics or bone or orthopaedic) and (surgery or surgeries or operation or operations or procedure or procedures or surgical)):ti,ab,kw 19,107

#3. MeSH descriptor: ["Orthopedic Procedures"] explode all trees 870

#4. MeSH descriptor: [Acetabuloplasty] explode all trees 7,961

#5. (((Orthopedic or Orthopaedic) and (Surgical or surgery) and (Procedures or Procedure)) or Acetabuloplasty or Arthrodesis or Arthroplasty or "Bone Lengthening" or "Bone Transplantation" or Cementoplasty or "Fracture Fixation" or Osteotomy or "Hip surgery" or "hip surgeries" or "Femur surgery" or "femur sugeries"):ti,ab,kw 14,856

#6. {or #1-#5} 28,221

#7. MeSH descriptor: ["blood transfusion"] explode all trees 3,306

#8. ("blood transfusion" or "blood transfusions" or transfusion or transfusions):ti,ab,kw 12,133

#9. #7 or #8 12,328

#10. [mh "intravenous injections"] or (intravenous or parenteral):ti,ab,kw 77,244

#11. MeSH descriptor: [Iron] explode all trees 4,228

#12. (iron or Ferritin or Ferritins or Isoferritin or "ferric compounds" or "ferric compound" or "ferrous compunds" or hemosiderin or transferrin):ti,ab,kw 8,814

#13. #11 or #12 9,856

#14. #6 and #9 and #10 and #13 41

KoreaMed (10-Sep-18)

((orthopedics [ALL] OR orthopedic [ALL] OR orthopaedic [ALL] OR orthopaedics [ALL]) AND (transfusion [ALL] OR transfusions [ALL]) AND (intravenous [ALL] OR parenteral [ALL]) AND (iron [ALL] OR ferritin [ALL] OR ferritins [ALL] OR isoferritin [ALL] OR transferrin [ALL])) 4

Search No. (Duplication No.)

PubMed	39 (0)	39
EMBASE	30 (22)	8
Cochrane	41 (15)	24
Koreamed	4 (1)	3
Google	1(0)	1
Total	115 (40)	
Final reference	75	
