# Supplementary material for: Efficacy of perioperative intravenous iron therapy for transfusion in orthopedic surgery: A systematic review and meta-analysis
Source: PLoS One. 2019 May 6;14(5):e0215427. doi: 10.1371/journal.pone.0215427 (PMC6502310; doi:10.1371/journal.pone.0215427)

Supporting Information

S 2 Fig. Egger's linear regression and Trim-and-fill analysis to assess publication bias

A. the ratio of patient transfused (%)

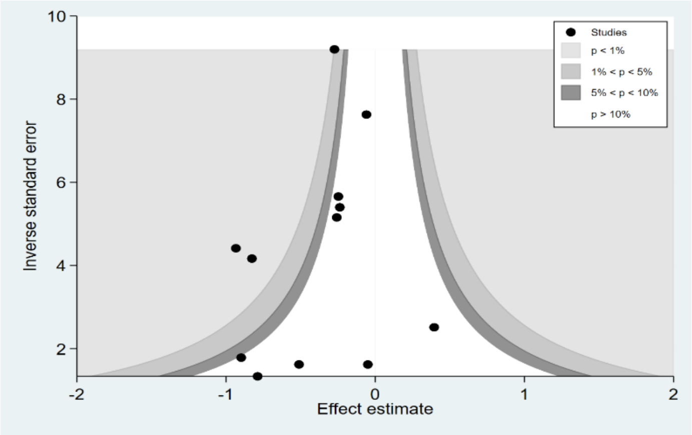

B. the units of RBC transfusion (U/patient)

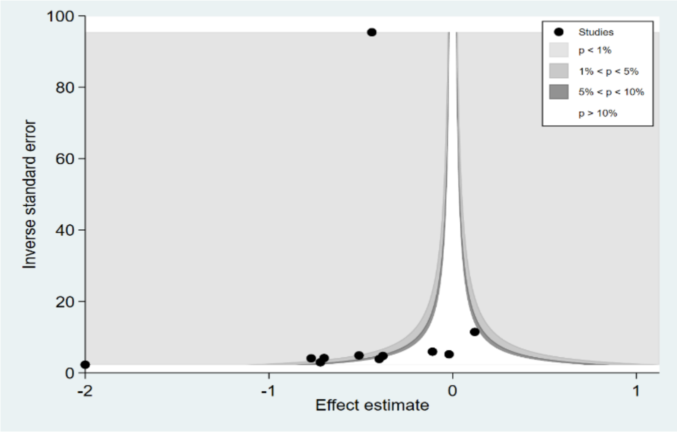

C. Length of hospital stay (days)

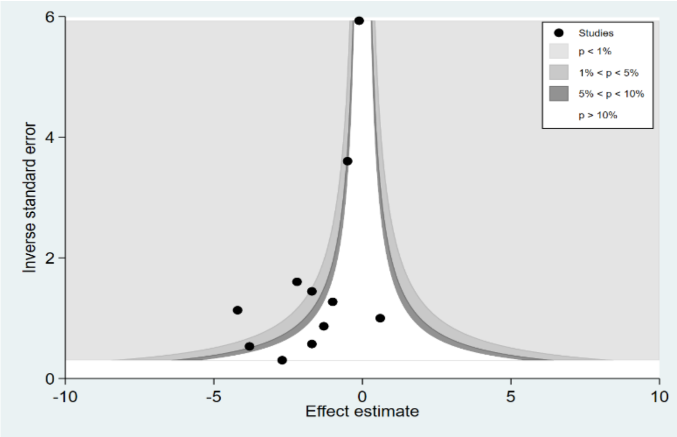

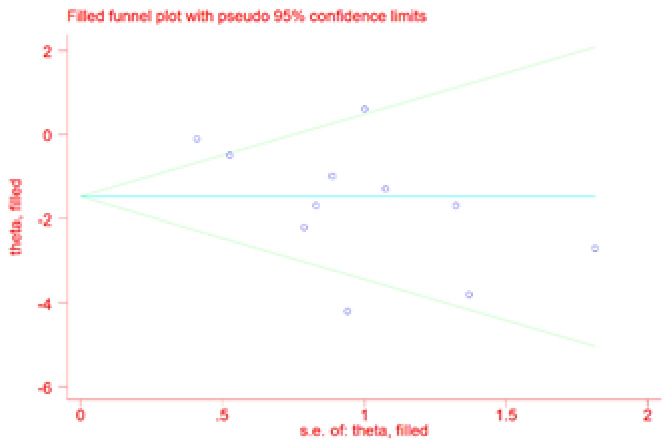

D. Postoperative infection (%)

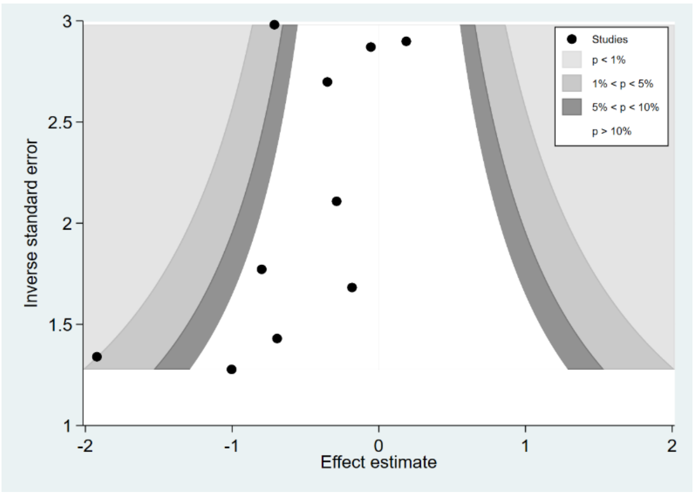

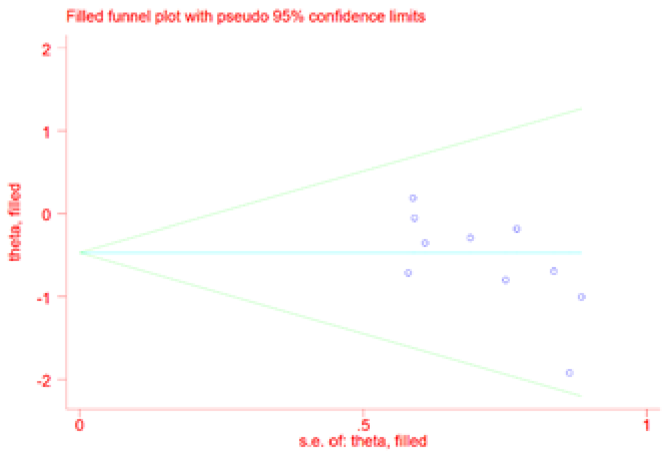

Supplement: S2 Fig — (PDF) [file pone.0215427.s002.pdf]
