# Supplementary material for: Efficacy of perioperative intravenous iron therapy for transfusion in orthopedic surgery: A systematic review and meta-analysis
Source: PLoS One. 2019 May 6;14(5):e0215427. doi: 10.1371/journal.pone.0215427 (PMC6502310; doi:10.1371/journal.pone.0215427)

Supporting Information

S 3 Fig. Randomized controlled trials (RCTs) vs. Case-controlled studies (CCSs)

A. Randomized controlled trials (RCTs) vs. Case-controlled studies (CCSs)_Patients transfused (%)

Study or Subgroup	IV_iron Events	IV_iron Total	Control Events	Control Total	Weight	Risk Ratio M-H, Random, 95% CI	Year
**19.1.1 RCTs**							
Karkouti 2006	2	11	4	10	1.6%	0.45 [0.10, 1.97]	2006
Serrano-Trenas 2011	33	99	41	97	11.4%	0.79 [0.55, 1.13]	2011
Bernabeu-Wittel 2016	53	101	54	97	14.2%	0.94 [0.73, 1.22]	2016
Khalafallah 2016	1	103	5	98	0.8%	0.19 [0.02, 1.60]	2016
**Subtotal (95% CI)**		**314**		**302**	**27.9%**	**0.84 [0.65, 1.09]**	
Total events	89		104				
Heterogeneity: Tau² = 0.01; Chi² = 3.54, df = 3 (P = 0.32); I² = 15%							
Test for overall effect: Z = 1.30 (P = 0.20)							
**19.1.2 CCSs**							
Cuenca 2004	24	55	57	102	11.8%	0.78 [0.55, 1.10]	2004
Cuenca 2005	3	20	21	57	2.7%	0.41 [0.14, 1.22]	2005
Munoz 2006	11	24	16	22	8.3%	0.63 [0.38, 1.04]	2006
Gonzalez-Porras 2009	10	49	11	80	4.6%	1.48 [0.68, 3.23]	2009
Bae 2010	17	30	22	30	10.9%	0.77 [0.53, 1.13]	2010
Blanco Rubio 2013	16	57	45	63	9.5%	0.39 [0.25, 0.61]	2013
Munoz 2014	21	182	48	182	8.9%	0.44 [0.27, 0.70]	2014
Kim 2018	70	150	92	150	15.4%	0.76 [0.61, 0.94]	2018
**Subtotal (95% CI)**		**567**		**686**	**72.1%**	**0.65 [0.51, 0.83]**	
Total events	172		312				
Heterogeneity: Tau² = 0.06; Chi² = 16.56, df = 7 (P = 0.02); I² = 58%							
Test for overall effect: Z = 3.49 (P = 0.0005)							
**Total (95% CI)**		**881**		**988**	**100.0%**	**0.69 [0.57, 0.84]**	
Total events	261		416				
Heterogeneity: Tau² = 0.05; Chi² = 23.56, df = 11 (P = 0.01); I² = 53%							
Test for overall effect: Z = 3.72 (P = 0.0002)							
Test for subgroup differences: Chi² = 2.01, df = 1 (P = 0.16), I² = 50.3%							

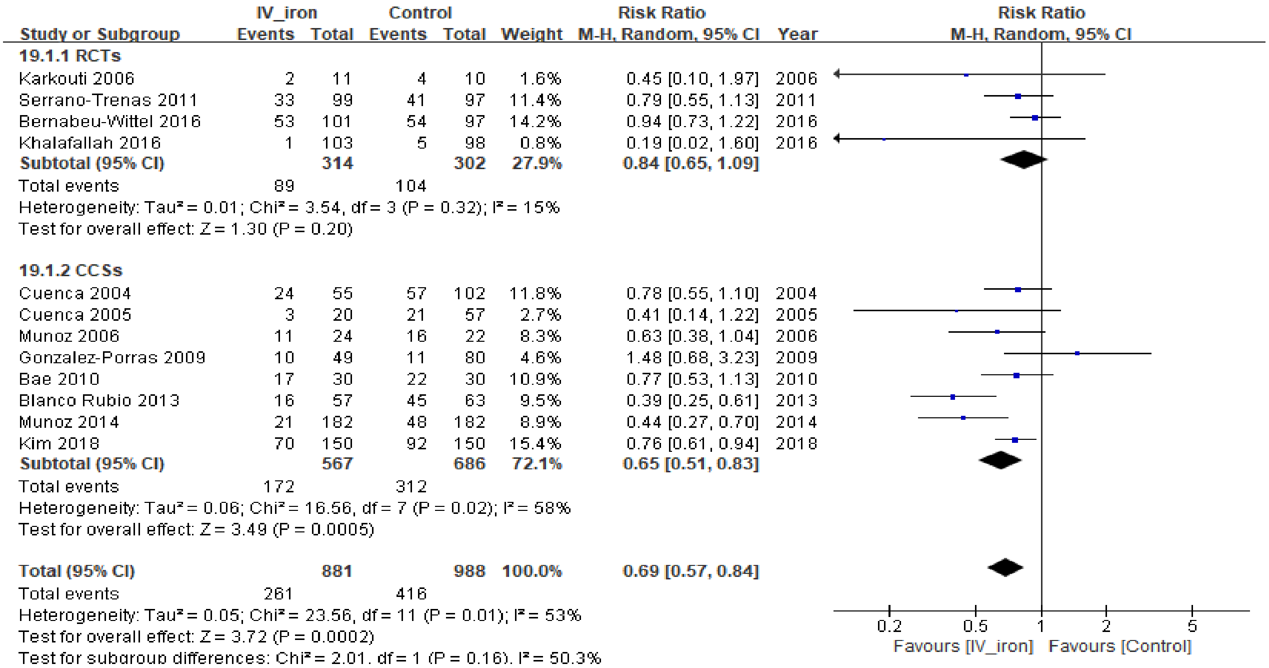

B. RCTs vs. CCSs_ RBCs units used by patients (unit/person)

Study or Subgroup	IV_iron Mean	IV_iron SD	IV_iron Total	Control Mean	Control SD	Control Total	Weight	Mean Difference IV, Random, 95% CI	Year
**20.1.1 RCTs**									
Karkouti 2006	0.27	0.5	11	0.5	0.5	10	8.4%	-0.23 [-0.66, 0.20]	2006
Serrano-Trenas 2011	0.76	1.16	99	0.87	1.2	97	10.1%	-0.11 [-0.44, 0.22]	2011
Bernabeu-Wittel 2016	1.26	1.3	101	1.28	1.4	97	9.3%	-0.02 [-0.40, 0.36]	2016
**Subtotal (95% CI)**			**211**			**204**	**27.7%**	**-0.11 [-0.33, 0.10]**	
Heterogeneity: Tau² = 0.00; Chi² = 0.52, df = 2 (P = 0.77); I² = 0%									
Test for overall effect: Z = 1.01 (P = 0.31)									
**20.1.2 CCSs**									
Cuenca 2004	0.89	1.22	55	1.27	1.34	102	8.6%	-0.38 [-0.79, 0.03]	2004
Cuenca 2005	0.26	0.65	20	0.77	1.09	57	8.8%	-0.51 [-0.91, -0.11]	2005
Munoz 2006	0.96	1.12	24	1.68	1.17	22	5.4%	-0.72 [-1.38, -0.06]	2006
Gonzalez-Porras 2009	0.37	0.52	49	0.25	0.41	80	12.7%	0.12 [-0.05, 0.29]	2009
Bae 2010	1.3	1	30	1.7	1	30	7.2%	-0.40 [-0.91, 0.11]	2010
Blanco Rubio 2013	0.6	1.193	57	1.37	1.495	63	7.6%	-0.77 [-1.25, -0.29]	2013
Munoz 2014	0.23	0.1	182	0.67	0.1	182	14.1%	-0.44 [-0.46, -0.42]	2014
Kim 2018	1	1.2	150	1.7	2.7	150	7.7%	-0.70 [-1.17, -0.23]	2018
**Subtotal (95% CI)**			**567**			**686**	**72.3%**	**-0.43 [-0.67, -0.19]**	
Heterogeneity: Tau² = 0.08; Chi² = 44.62, df = 7 (P < 0.00001); I² = 84%									
Test for overall effect: Z = 3.46 (P = 0.0005)									
**Total (95% CI)**			**778**			**890**	**100.0%**	**-0.34 [-0.53, -0.14]**	
Heterogeneity: Tau² = 0.07; Chi² = 53.72, df = 10 (P < 0.00001); I² = 81%									
Test for overall effect: Z = 3.39 (P = 0.0007)									
Test for subgroup differences: Chi² = 3.66, df = 1 (P = 0.06), I² = 72.7%									

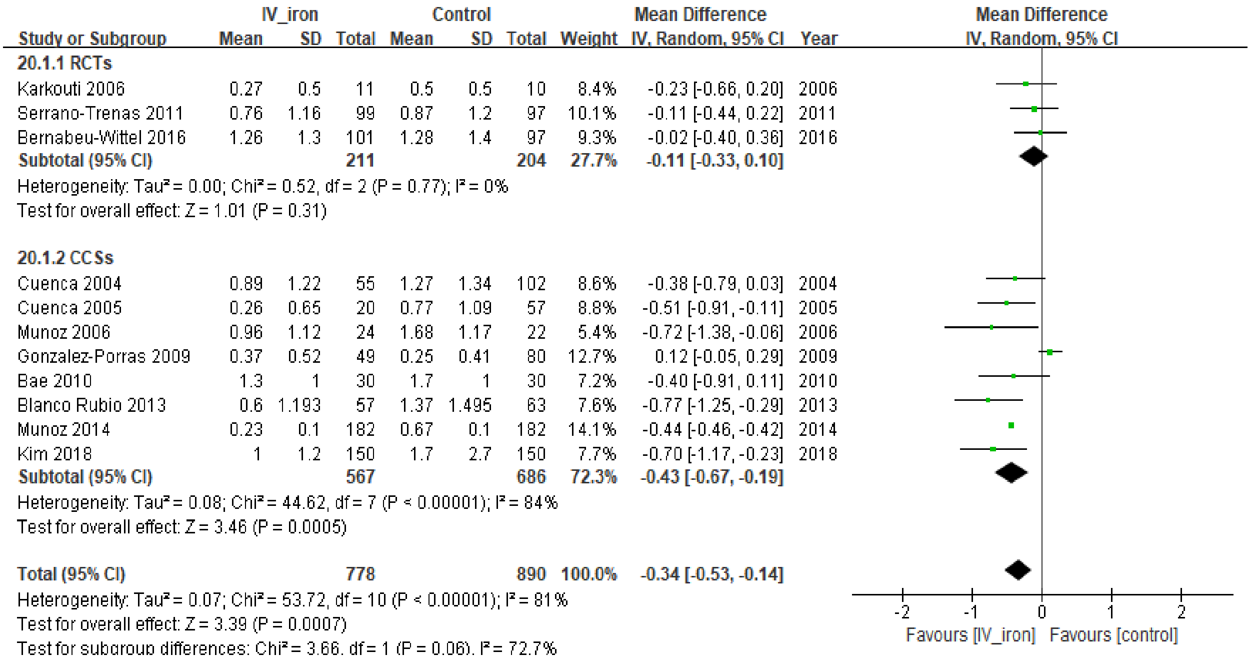

C. CTs vs. CCSs_ Length of hospital stay (days)

Study or Subgroup	IV_iron Mean	IV_iron SD	IV_iron Total	Control Mean	Control SD	Control Total	Weight	Mean Difference IV, Random, 95% CI	Year
**21.1.1 RCTs**									
Karkouti 2006	12.6	4.4	11	14.3	3.6	10	5.2%	-1.70 [-5.13, 1.73]	2006
Serrano-Trenas 2011	13.5	7.1	99	12.9	6.9	97	10.2%	0.60 [-1.36, 2.56]	2011
Bernabeu-Wittel 2016	7	5	101	8	6	97	12.4%	-1.00 [-2.54, 0.54]	2016
Khalafallah 2016	7.8	10.3	103	11.6	15.6	98	4.7%	-3.80 [-7.47, -0.13]	2016
**Subtotal (95% CI)**			**314**			**302**	**32.5%**	**-0.98 [-2.50, 0.54]**	
Heterogeneity: Tau² = 0.90; Chi² = 4.85, df = 3 (P = 0.18); I² = 38%									
Test for overall effect: Z = 1.27 (P = 0.21)									
**21.1.2 CCSs**									
Cuenca 2004	12.6	4.4	55	14.3	3.6	102	13.4%	-1.70 [-3.06, -0.34]	2004
Cuenca 2005	11.9	2.1	20	14.1	3.1	57	14.2%	-2.20 [-3.42, -0.98]	2005
Munoz 2006	10.1	4.4	24	11.4	3.4	22	8.8%	-1.30 [-3.56, 0.96]	2006
Bae 2010	17.5	10	30	20.2	15	30	1.8%	-2.70 [-9.15, 3.75]	2010
Munoz 2014	7.9	2.1	182	8.4	3.1	182	17.8%	-0.50 [-1.04, 0.04]	2014
Kim 2018	7.6	3.3	150	11.8	10.3	150	11.4%	-4.20 [-5.93, -2.47]	2018
**Subtotal (95% CI)**			**461**			**543**	**67.5%**	**-1.91 [-3.15, -0.68]**	
Heterogeneity: Tau² = 1.52; Chi² = 21.11, df = 5 (P = 0.0008); I² = 76%									
Test for overall effect: Z = 3.03 (P = 0.002)									
**Total (95% CI)**			**775**			**845**	**100.0%**	**-1.60 [-2.52, -0.68]**	
Heterogeneity: Tau² = 1.15; Chi² = 26.22, df = 9 (P = 0.002); I² = 66%									
Test for overall effect: Z = 3.42 (P = 0.0006)									
Test for subgroup differences: Chi² = 0.87, df = 1 (P = 0.35), I² = 0%									

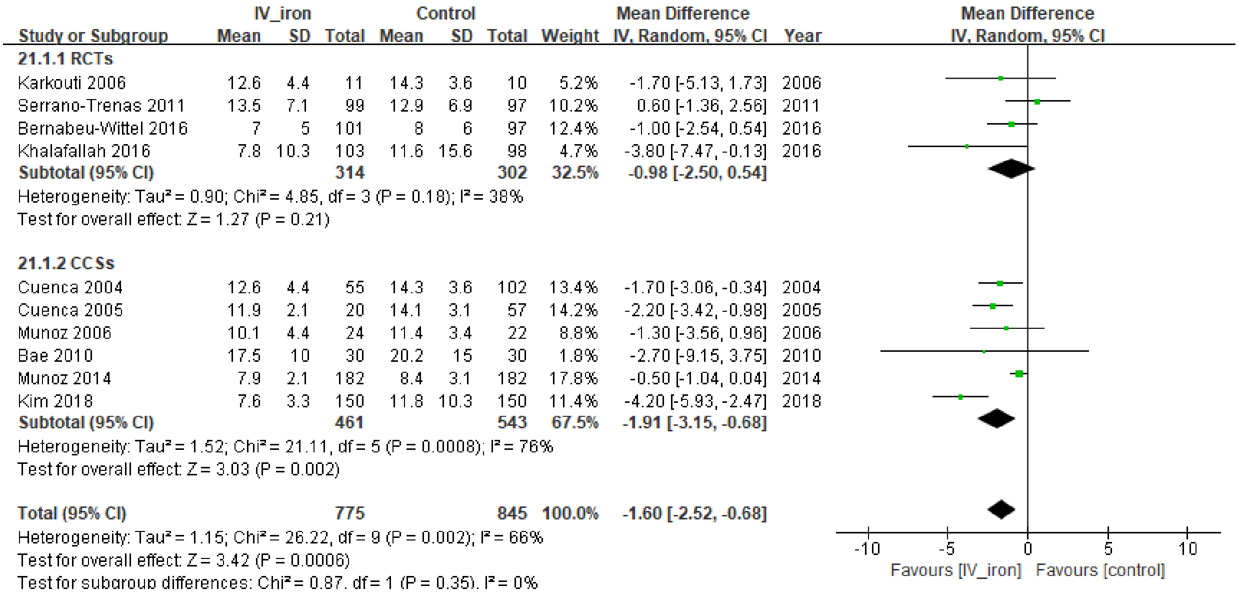

D. RCTs vs. CCSs_ Postoperative infection (%)

Study or Subgroup	IV_iron Events	IV_iron Total	Control Events	Control Total	Weight	Risk Ratio M-H, Random, 95% CI	Year
**22.1.1 RCTs**							
Serrano-Trenas 2011	16	99	13	97	16.3%	1.21 [0.61, 2.37]	2011
Khalafallah 2016	2	103	13	98	4.3%	0.15 [0.03, 0.63]	2016
Bernabeu-Wittel 2016	11	101	15	97	14.5%	0.70 [0.34, 1.46]	2016
**Subtotal (95% CI)**		**303**		**292**	**35.0%**	**0.61 [0.23, 1.58]**	
Total events	29		41				
Heterogeneity: Tau² = 0.49; Chi² = 6.98, df = 2 (P = 0.03); I² = 71%							
Test for overall effect: Z = 1.02 (P = 0.31)							
**22.1.2 CCSs**							
Cuenca 2004	9	55	34	102	17.0%	0.49 [0.25, 0.95]	2004
Cuenca 2005	3	20	19	57	7.1%	0.45 [0.15, 1.36]	2005
Munoz 2006	2	24	5	22	3.9%	0.37 [0.08, 1.70]	2006
Bae 2010	6	30	8	30	9.7%	0.75 [0.30, 1.90]	2010
Blanco Rubio 2013	12	57	14	63	16.0%	0.95 [0.48, 1.88]	2013
Munoz 2014	3	182	6	182	4.8%	0.50 [0.13, 1.97]	2014
Kim 2018	5	150	6	150	6.5%	0.83 [0.26, 2.67]	2018
**Subtotal (95% CI)**		**518**		**606**	**65.0%**	**0.64 [0.45, 0.90]**	
Total events	40		92				
Heterogeneity: Tau² = 0.00; Chi² = 3.25, df = 6 (P = 0.78); I² = 0%							
Test for overall effect: Z = 2.53 (P = 0.01)							
**Total (95% CI)**		**821**		**898**	**100.0%**	**0.67 [0.49, 0.91]**	
Total events	69		133				
Heterogeneity: Tau² = 0.04; Chi² = 10.55, df = 9 (P = 0.31); I² = 15%							
Test for overall effect: Z = 2.52 (P = 0.01)							
Test for subgroup differences: Chi² = 0.01, df = 1 (P = 0.93), I² = 0%							

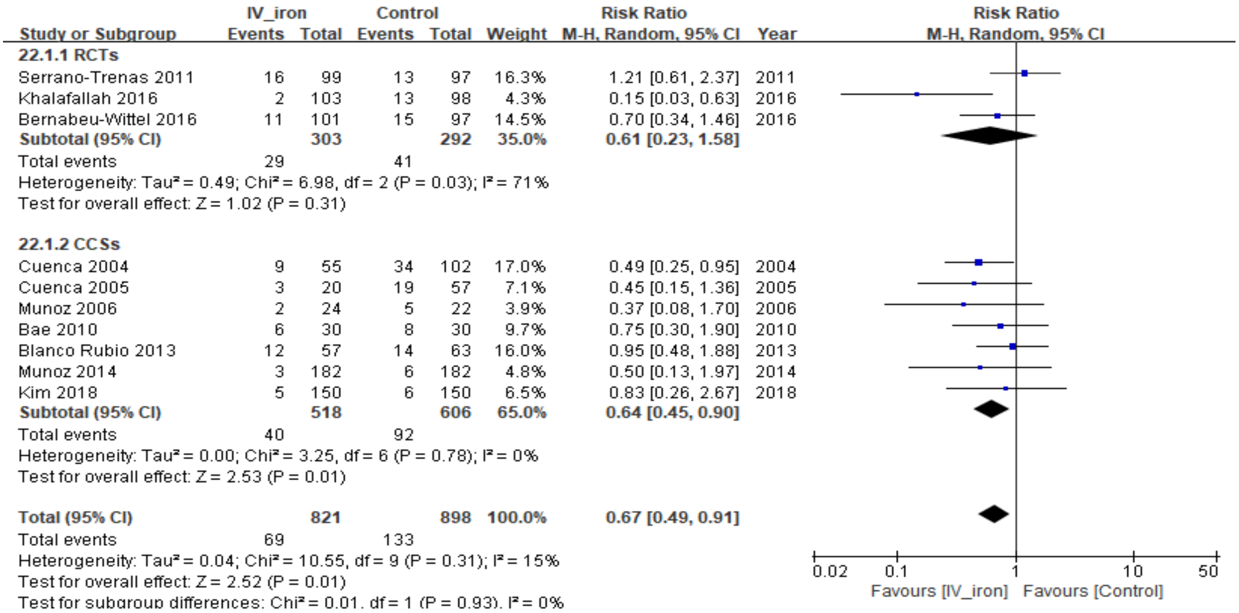

E. RCTs vs. CCSs_ Mortality (%)

Study or Subgroup	IV_iron Events	IV_iron Total	Control Events	Control Total	Weight	Risk Ratio M-H, Random, 95% CI	Year
**5.1.1 RCTs_Mortality (%)**							
Serrano-Trenas 2011	11	99	10	97	22.5%	1.08 [0.48, 2.42]	2011
Bernabeu-Wittel 2016	3	101	28	97	20.2%	0.10 [0.03, 0.33]	2016
**Subtotal (95% CI)**		**200**		**194**	**42.7%**	**0.34 [0.03, 3.81]**	
Total events	14		38				
Heterogeneity: Tau² = 2.75; Chi² = 11.59, df = 1 (P = 0.0007); I² = 91%							
Test for overall effect: Z = 0.87 (P = 0.39)							
**5.1.2 CCSs_Mortality (%)**							
Cuenca 2004	5	55	3	102	18.6%	3.09 [0.77, 12.45]	2004
Cuenca 2005	0	20	11	57	10.3%	0.12 [0.01, 1.95]	2005
Munoz 2006	0	24	1	22	8.9%	0.31 [0.01, 7.16]	2006
Blanco Rubio 2013	4	57	5	63	19.5%	0.88 [0.25, 3.13]	2013
**Subtotal (95% CI)**		**156**		**244**	**57.3%**	**0.86 [0.23, 3.19]**	
Total events	9		20				
Heterogeneity: Tau² = 0.81; Chi² = 5.70, df = 3 (P = 0.13); I² = 47%							
Test for overall effect: Z = 0.23 (P = 0.82)							
**Total (95% CI)**		**356**		**438**	**100.0%**	**0.56 [0.17, 1.79]**	
Total events	23		58				
Heterogeneity: Tau² = 1.40; Chi² = 19.35, df = 5 (P = 0.002); I² = 74%							
Test for overall effect: Z = 0.98 (P = 0.33)							
Test for subgroup differences: Chi² = 0.43, df = 1 (P = 0.51), I² = 0%							

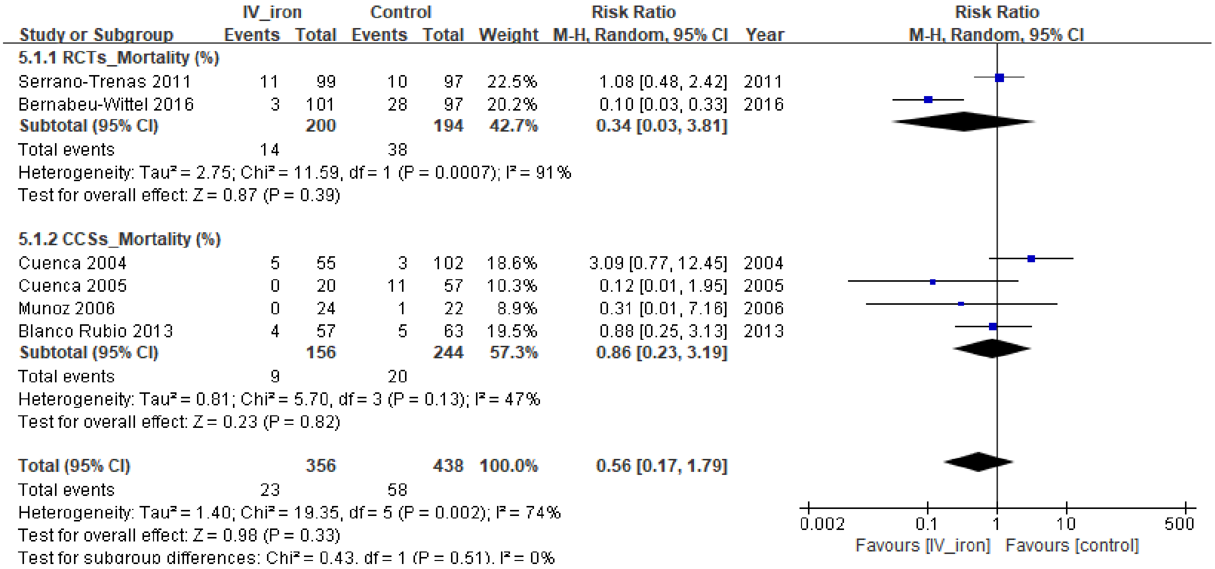

Supplement: S3 Fig — (PDF) [file pone.0215427.s003.pdf]
