# Supplementary material for: Efficacy of perioperative intravenous iron therapy for transfusion in orthopedic surgery: A systematic review and meta-analysis
Source: PLoS One. 2019 May 6;14(5):e0215427. doi: 10.1371/journal.pone.0215427 (PMC6502310; doi:10.1371/journal.pone.0215427)

Supporting Information

S 4 Fig.

A. The comparison of hemoglobin (Hb) level between IVIT and control groups

① Hb level at admission

Study or Subgroup	IV_iron Mean	IV_iron SD	IV_iron Total	Control Mean	Control SD	Control Total	Weight	Mean Difference IV, Random, 95% CI	Year
Cuenca 2004	12.4	2.2	55	12.3	1.8	102	8.8%	0.10 [-0.58, 0.78]	2004
Cuenca 2005	12.4	1.2	20	12.5	1.4	57	8.9%	-0.10 [-0.74, 0.54]	2005
Munoz 2006	12.2	2	24	12.8	2	22	7.2%	-0.60 [-1.76, 0.56]	2006
Karkouti 2006	14	1.1	13	14.2	0.9	13	8.5%	-0.20 [-0.97, 0.57]	2006
Gonzalez-Porras 2009	13.3	0.2	49	14.9	0.4	80	9.9%	-1.60 [-1.70, -1.50]	2009
Bae 2010	12.2	1.5	30	12.7	1.5	30	8.6%	-0.50 [-1.26, 0.26]	2010
Serrano-Trenas 2011	12.1	1.6	99	11.9	1.8	97	9.3%	0.20 [-0.28, 0.68]	2011
Munoz 2014	13.8	1.2	182	13.6	1.2	182	9.8%	0.20 [-0.05, 0.45]	2014
Khalafallah 2016	13.4	1.3	103	13.4	1.3	98	9.6%	0.00 [-0.36, 0.36]	2016
Bernabeu-Wittel 2016	10.9	0.7	103	11	0.7	100	9.8%	-0.10 [-0.29, 0.09]	2016
Kim 2018	12.2	1.5	150	12.5	1.8	150	9.6%	-0.30 [-0.67, 0.07]	2018
**Total (95% CI)**			**828**			**931**	**100.0%**	**-0.26 [-0.86, 0.34]**	

Heterogeneity: Tau² = 0.94; Chi² = 389.71, df = 10 (P < 0.00001); I² = 97%
Test for overall effect: Z = 0.86 (P = 0.39)

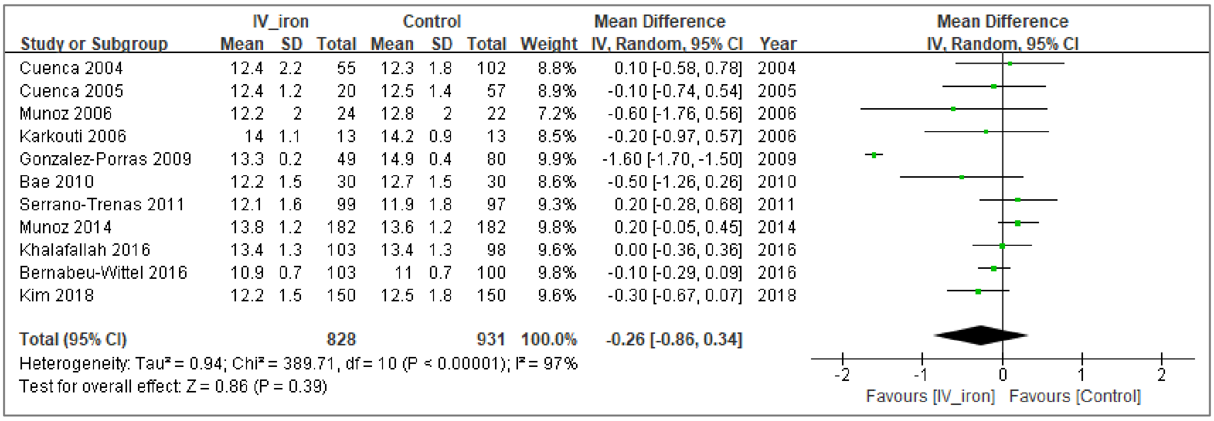

② Hb level on postoperative day 1

Study or Subgroup	IV_iron Mean	IV_iron SD	IV_iron Total	Control Mean	Control SD	Control Total	Weight	Mean Difference IV, Random, 95% CI	Year
Cuenca 2005	9.6	3	20	9.4	2	57	6.2%	0.20 [-1.21, 1.61]	2005
Munoz 2006	9.8	0.5	24	10	0.5	22	19.3%	-0.20 [-0.49, 0.09]	2006
Karkouti 2006	8.5	0.4	13	8.3	0.4	13	19.1%	0.20 [-0.11, 0.51]	2006
Bernabeu-Wittel 2016	9.2	1.2	103	9.3	1.2	100	18.8%	-0.10 [-0.43, 0.23]	2016
Khalafallah 2016	10.6	1.1	103	10.5	1.3	98	18.7%	0.10 [-0.23, 0.43]	2016
Kim 2018	9.5	1.7	150	10.7	1.7	150	18.0%	-1.20 [-1.58, -0.82]	2018
**Total (95% CI)**			**413**			**440**	**100.0%**	**-0.20 [-0.62, 0.21]**	

Heterogeneity: Tau² = 0.21; Chi² = 36.08, df = 5 (P < 0.00001); I² = 86%
Test for overall effect: Z = 0.96 (P = 0.34)

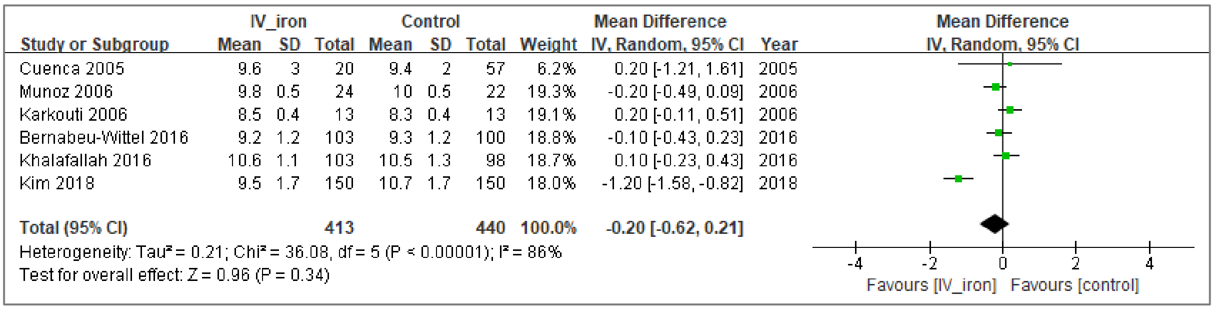

③ Hb level on postoperative day 7

Study or Subgroup	IV-iron Mean	IV-iron SD	IV-iron Total	Control Mean	Control SD	Control Total	Weight	Mean Difference IV, Random, 95% CI	Year
Munoz 2006	9.5	1	24	10	1	22	15.7%	-0.50 [-1.08, 0.08]	2006
Karkouti 2006	9.7	0.7	13	9.6	0.8	13	15.8%	0.10 [-0.48, 0.68]	2006
Bae 2010	10.4	1	30	10.2	1	30	16.2%	0.20 [-0.31, 0.71]	2010
Munoz 2014	10.6	1.6	182	9.2	2.2	182	16.9%	1.40 [1.00, 1.80]	2014
Bernabeu-Wittel 2016	10	1	103	9.7	0.1	100	17.8%	0.30 [0.11, 0.49]	2016
Kim 2018	9.3	1.1	150	10.1	1.2	150	17.6%	-0.80 [-1.06, -0.54]	2018
**Total (95% CI)**			**502**			**497**	**100.0%**	**0.12 [-0.52, 0.76]**	

Heterogeneity: Tau² = 0.59; Chi² = 95.70, df = 5 (P < 0.00001); I² = 95%
Test for overall effect: Z = 0.37 (P = 0.71)

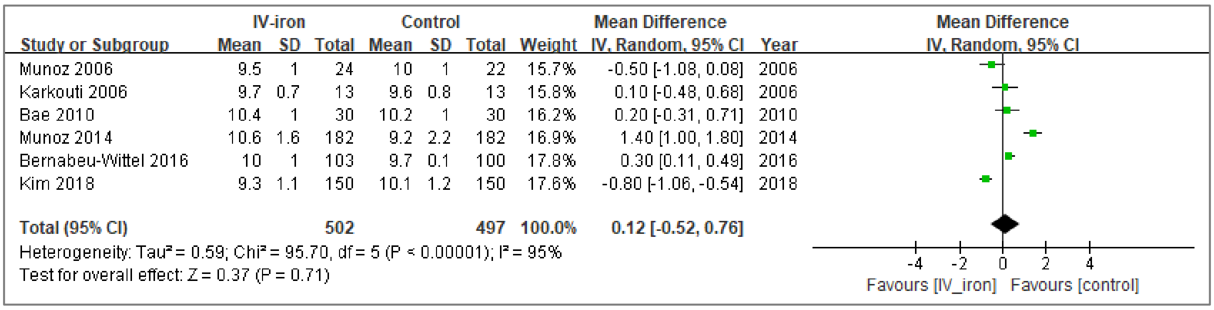

④ Hb level at 6 weeks or 60 days or 12 weeks postoperatively

Study or Subgroup	Experimental Mean	Experimental SD	Experimental Total	Control Mean	Control SD	Control Total	Weight	Mean Difference IV, Random, 95% CI	Year
Karkouti 2006	12.7	0.6	13	12	1.3	13	16.7%	0.70 [-0.08, 1.48]	2006
Gonzalez-Porras 2009	10.3	0.8	49	11.1	0.8	80	16.7%	-0.80 [-1.08, -0.52]	2009
Khalafallah 2016	13.7	1.1	103	13.3	1.1	98	16.7%	0.40 [0.10, 0.70]	2016
Bernabeu-Wittel 2016	12.8	1.5	103	11.9	1.1	100	16.7%	0.90 [0.54, 1.26]	2016
Khalafallah 2016	143.1	1.1	103	12.1	1.4	98	16.7%	131.00 [130.65, 131.35]	2016
Kim 2018	12.5	1.4	150	12.1	1.5	150	16.7%	0.40 [0.07, 0.73]	2018
**Total (95% CI)**			**521**			**539**	**100.0%**	**22.10 [-21.10, 65.30]**	

Heterogeneity: Tau² = 2914.93; Chi² = 450868.52, df = 5 (P < 0.00001); I² = 100%
Test for overall effect: Z = 1.00 (P = 0.32)

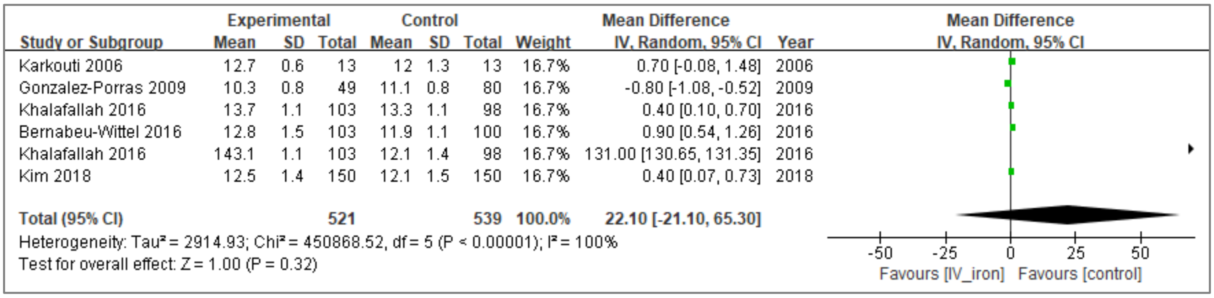

S 4 Fig.

B. Comparison of ferritin levels between IVIT and control groups

① Ferritin level at baseline or admission

Study or Subgroup	IV_iron Mean	IV_iron SD	IV_iron Total	Control Mean	Control SD	Control Total	Weight	Mean Difference IV, Random, 95% CI
Gonzalez-Porras 2009	127	227	49	129	126	80	42.2%	-2.00 [-71.30, 67.30]
Khalafallah 2016	118	209	103	188	103	98	57.8%	-70.00 [-115.22, -24.78]
**Total (95% CI)**			**152**			**178**	**100.0%**	**-41.28 [-107.11, 24.55]**

Heterogeneity: Tau² = 1420.80; Chi² = 2.59, df = 1 (P = 0.11); I² = 61%
Test for overall effect: Z = 1.23 (P = 0.22)

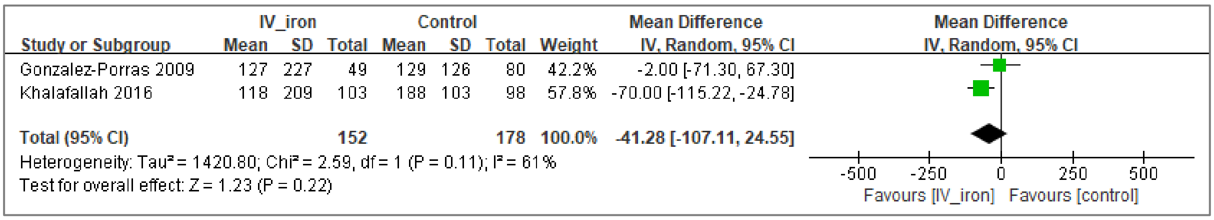

② Ferritin level on postoperative day 1

Study or Subgroup	IV_iron Mean	IV_iron SD	IV_iron Total	Control Mean	Control SD	Control Total	Weight	Mean Difference IV, Random, 95% CI
Karkouti 2006	258	224	11	259	174	10	16.3%	-1.00 [-171.74, 169.74]
Khalafallah 2016	304	186	103	329	335	98	83.7%	-25.00 [-100.43, 50.43]
**Total (95% CI)**			**114**			**108**	**100.0%**	**-21.08 [-90.08, 47.91]**

Heterogeneity: Tau² = 0.00; Chi² = 0.06, df = 1 (P = 0.80); I² = 0%
Test for overall effect: Z = 0.60 (P = 0.55)

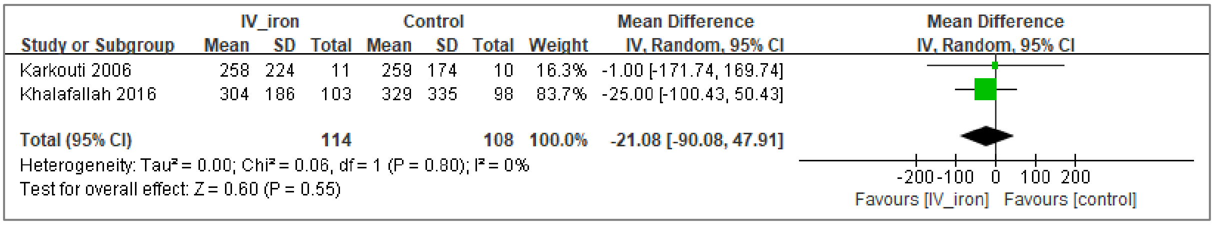

③ Ferritin level on postoperative day 7

Study or Subgroup	IV_iron Mean	IV_iron SD	IV_iron Total	Control Mean	Control SD	Control Total	Weight	Mean Difference IV, Random, 95% CI
Bernabeau_Wittel 2016	1,067	170	101	249	250	97	51.5%	818.00 [758.21, 877.79]
Karkouti 2006	513	221	11	311	286	10	48.5%	202.00 [-18.18, 422.18]
**Total (95% CI)**			**112**			**107**	**100.0%**	**519.49 [-83.89, 1122.87]**

Heterogeneity: Tau² = 182952.92; Chi² = 28.00, df = 1 (P < 0.00001); I² = 96%
Test for overall effect: Z = 1.69 (P = 0.09)

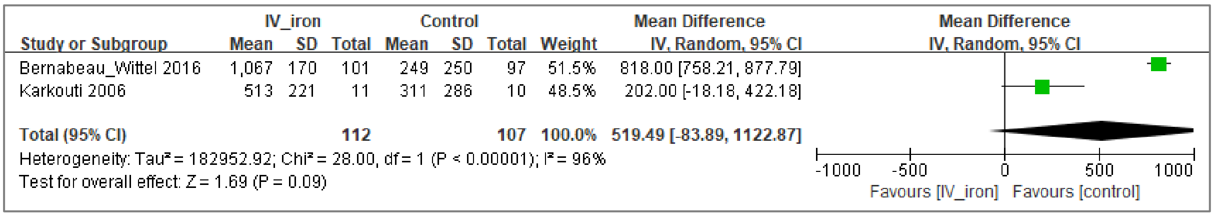

④ Ferritin level at 4 weeks or 60days postoperatively

Study or Subgroup	IV_iron Mean	IV_iron SD	IV_iron Total	Control Mean	Control SD	Control Total	Weight	Mean Difference IV, Random, 95% CI
Bernabeau_Wittel 2016	472	310	101	137	120	97	55.9%	335.00 [270.00, 400.00]
Khalafallah 2016	717	403	103	274	296	98	44.1%	443.00 [345.58, 540.42]
**Total (95% CI)**			**204**			**195**	**100.0%**	**382.65 [277.55, 487.76]**

Heterogeneity: Tau² = 4046.62; Chi² = 3.27, df = 1 (P = 0.07); I² = 69%
Test for overall effect: Z = 7.14 (P < 0.00001)

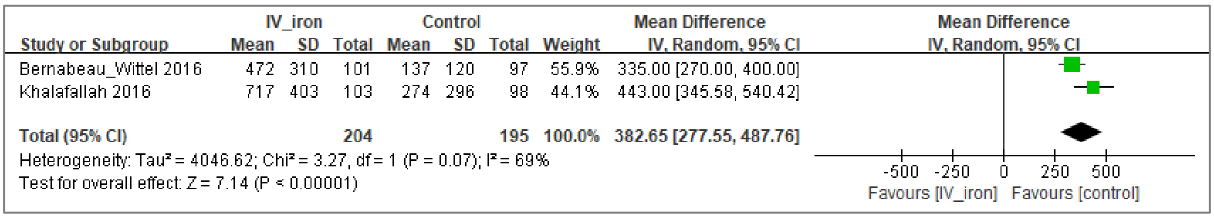

Supplement: S4 Fig — (PDF) [file pone.0215427.s004.pdf]
